# Long noncoding RNA *Neat1* modulates myogenesis by recruiting Ezh2

**DOI:** 10.1038/s41419-019-1742-7

**Published:** 2019-06-26

**Authors:** Shanshan Wang, Hao Zuo, Jianjun Jin, Wei Lv, Zaiyan Xu, Yonghui Fan, Jiali Zhang, Bo Zuo

**Affiliations:** 1 Key Laboratory of Swine Genetics and Breeding of the Ministry of Agriculture and Rural Affairs, Huazhong Agricultural University, 430070 Wuhan, Hubei People’s Republic of China; 2 Key Laboratory of Agriculture Animal Genetics, Breeding and Reproduction of the Ministry of Education, Huazhong Agricultural University, 430070 Wuhan, Hubei People’s Republic of China; 3 Department of Basic Veterinary Medicine, College of Veterinary Medicine, Huazhong Agricultural University, 430070 Wuhan, Hubei People’s Republic of China; 4 The Cooperative Innovation Center for Sustainable Pig Production, 430070 Wuhan, People’s Republic of China

**Keywords:** Gene silencing, Gene silencing, Gene silencing, Long non-coding RNAs, Long non-coding RNAs

## Abstract

*Neat1* is widely expressed in many tissues and cells and exerts pro-proliferation effects on many cancer cells. However, little is known about the function of *Neat1* in myogenesis. Here we characterized the roles of *Neat1* in muscle cell formation and muscle regeneration. Gain- or loss-of-function studies in C2C12 cells demonstrated that *Neat1* accelerates myoblast proliferation but suppresses myoblast differentiation and fusion. Further, knockdown of Neat1 in vivo increased the cross-sectional area of muscle fibers but impaired muscle regeneration. Mechanically, *Neat1* physically interacted with Ezh2 mainly through the core binding region (1001–1540 bp) and recruited Ezh2 to target gene promoters. *Neat1* promoted myoblast proliferation mainly by decreasing the expression of the cyclin-dependent kinase inhibitor *P21* gene but inhibited myoblast differentiation by suppressing the transcription of myogenic marker genes, such as *Myog*, *Myh4*, and *Tnni2*. Altogether, we uncover a previously unknown function of *Neat1* in muscle development and the molecular mechanism by which *Neat1* regulates myogenesis.

## Introduction

Skeletal muscle is the most abundant tissue in the mammalian body and plays a pivotal role in regulating body metabolism and homeostasis^1^. The differentiation of skeletal muscle cells is precisely regulated by several myogenic regulatory factors (MRFs), including myogenic differentiation 1 (*Myod*), myogenic factor 5 (*Myf5*), myogenin (*Myog*), and *Mrf4*^2^. *Myod* or *Myf5* is necessary for skeletal muscle lineage formation and is expressed at the myoblast stage^3^. *Myod* overexpression converts fibroblasts into myoblasts and subsequent fusion into myotubes^4,5^. *Myog* and *Mrf4* are expressed after *Myod* and *Myf5* and determine terminal muscle cell differentiation. *Myog* knockdown reversed terminal muscle cell differentiation^6^. MRFs also contribute to the regeneration of injured adult muscle, as muscle regeneration demands activation of the muscle regulatory network^7,8^. During injury, satellite cells (SCs) are activated and undergoing proliferation, and paired box (*Pax*) *7* and *Myod* genes are upregulated at this stage. Next, SCs differentiate into myotubes, upon which *Pax* genes are downregulated and *Myog* upregulated^9^. Epigenetic regulation, such as DNA methylation^10^, histone modifications^11,12^, and noncoding RNA functions^13,14^, also play important roles in the transcriptional regulation of myogenesis and ensure the normal proliferation and differentiation of muscle progenitors^15,16^. Enhancer of zeste homolog 2 (Ezh2) is a subunit of the epigenetic regulator polycomb repressive complex 2 (PRC2) responsible for trimethylation of lysine 27 of histone 3 (H3k27me3), which leads to repression of gene transcription. A previous study established the important role of polycomb-mediated H3k27 methylation during myogenic differentiation^17^. Ezh2 overexpression suppresses myogenic differentiation by silencing muscle-specific genes^18,19^. Long non-coding RNAs (lncRNAs) (e.g., Linc-*MD1*^20^, Lnc-*mg*^21^, LncRNA-*YY1*^22^, Linc-*RAM*^23^, *Myolinc*^24^, *MAR1*^25^, *AK017368*^26^, *SYISL*^27^), which are greater than 200 nucleotides in length and have no protein-coding capacity, were recently reported to play important roles in myogenesis by interacting with various proteins or acting as molecular sponges for miRNAs.

Nuclear paraspeckle assembly transcript 1 (*NEAT1*, known as *Neat1* in mouse) is a lncRNA that is enriched in the nucleus and essential for nuclear paraspeckle formation^28,29^. Paraspeckles were recently identified as mammalian-specific nuclear bodies that are found in most cells cultured in vitro but are not essential in vivo^30^, Paraspeckles play important roles in many gene regulation processes, such as mRNA retention, A-to-I editing, and protein sequestration^31,32^. *NEAT1* serves as a platform to recruit numerous paraspeckle proteins to maintain paraspeckle stability and integrity^32–34^. In addition, long-range interactions among *NEAT1* transcripts may exert an important architectural function in paraspeckles formation^35^. In addition to participating in the formation of paraspeckles, *NEAT1* also plays important roles in a variety of biological processes. For example, *NEAT1* regulates the phenotypic switch of vascular smooth muscle cells by inhibiting SM (smooth muscle)-contractile gene expression by removing the epigenetic activator WDR5 from SM-specific gene loci^36^. *NEAT1* is widely expressed in multiple tissues and participates in the tumorigenesis of many cancers including prostate cancer^37^, breast cancer^38^, colorectal cancer^39^, esophageal squamous cell carcinoma^40^, laryngeal squamous cell cancer^41^, and pancreatic cancer^42^. Despite the important roles of *Neat1* in regulating multiple biological processes, it is unknown whether it is involved in muscle development and regeneration. In the present study, we investigated the roles of *Neat1* in myogenesis and found that *Neat1* regulates myoblast proliferation and differentiation by interacting with Ezh2, defining a novel function of *Neat1* in muscle development and regeneration.

## Materials and methods

### Cell culture

Mouse C2C12 cells were cultured in DMEM (high-glucose Dulbecco’s modified Eagle’s medium) (Hyclone, USA) containing 10% fetal bovine serum (Gibco, Australia) under moist air with 5% CO_2_ at 37 °C for proliferation and in DMEM with 2% horse serum (Gibco, USA) at the same condition for differentiation.

### Animals

C57 mice were purchased from Hubei center for disease control and housed in Huazhong Agricultural University under normal conditions with appropriate temperature and humidity and supplied with nutritional food and sufficient water. Animal feeding and tests were conducted based on the National Research Council Guide for the Care and Use of Laboratory Animals and approved by the Institutional Animal Care and Use Committee at Huazhong Agricultural University.

### Plasmid construction, siRNA synthesis

The full-length sequence of *Neat1*, and coding sequences (CDS) of *Ezh2* and *P21* were amplified by polymerase chain reaction (PCR) with corresponding full-length or cds F/R primers using C2C12 cDNA as a template. The amplified sequences were cloned into pcDNA3.1 using T4 DNA ligase (Takara,Japan) to produce pcDNA3.1*-Neat1*, pcDNA3.1*-Ezh2* and pcDNA3.1*-P21*. The truncated *Neat1* were obtained by PCR using pcDNA3.1*- Neat1* plasmid as a template and then were cloned into pcDNA3.1. The plasmids were confirmed by sequencing. The primers above were shown at Supplementary Table S1. siRNA oligos against mouse *Neat1* (sense 5′- GGAGUCAUGCCUUAUACAATT-3′), *Ezh2* (sense 5′- GCGCAGUAGAAUGGAGAAATT-3′) and *P21* (sense 5′-UGAGCAAUGGCUGAUCCUU-3′) were designed and synthesized by GenePharma (China, Shanghai).

### Transfection of plasmid, siRNA

For cell transfection, expression plasmids or siRNAs were conducted with Lipofectamine 2000 (Invitrogen, USA) as advised by the manufacturer’s protocol.

### Quantitative real-time PCR

RNA samples from C2C12 cells or mice tissues were isolated using the TRIzol reagent (Invitrogen, USA). The expression of mRNA was detected by Quantitative real-time PCR (qPCR). The qPCR reaction was performed in LightCycler 480 II (Roche, Switzerland) system using SYBR^®^Green Real-time PCR Master Mix (Toyobo, Japan). All the experiments were designed in triplicates. The relative gene expression was calculated by the Ct (2^-ΔΔCt^) method according to the literature^43^. The sequence primers were list at Supplementary Table S2.

### Cell proliferation assays

For real-time cell proliferation monitoring assay, C2C12 cells were inoculated on a 16-well E-Plate and transfected with *Neat1* expression vector or si*Neat1* oligos. The cell proliferation rates were recored by the RTCA xCELLigence system (Roche Applied Science, Penzberg, Upper Bavaria, Germany).

For EdU staining, the EdU staining was performed using EDU kit (RiboBio, China) according to the manufacturer’s instructions. Images were captured with an Olympus IX51-A21PH fluorescence microscope (Olympus, Japan). Cells were further analyzed by computing the percentage of EdU^+^ cells.

For EdU-propidium iodide (PI) flow cytometry, Edu reagent was added to C2C12 cells at a final concentration of 50 uM and incubated for 30 min at 37℃. Then the cells were harvested and fixed in 70% ethanol at 4 ℃ overnight. The cells were further carried out with EdU staining using EdU kit (RiboBio, China) according to the manufacturer’s instructions. After that the cells were incubated in 50 mg/ml PI for 1 h at room temperature, cells were analyzed using the FACSCalibur Flow Cytometer (Becton Dickinson, Franklin Lakes, NJ, USA).

### Western blotting

C2C12 cells or mouse tissues were lysed in RIPA buffer containing 1% (v/v) phenylmethylsulfonyl fluoride (PMSF) (Beyotime, Jiangsu, China). The western blotting was performed according to the previous publication^44^. The antibodies and their dilutions were shown as following: Myod (Santa Cruz Biotechnology, USA; sc-760; 1:1000), Myog (Santa Cruz Biotechnology, USA; sc-12732; 1:200), Myhc (Santa Cruz Biotechnology, USA; sc-376157; 1:3000), α-actin (Proteintech, China; 23660-1-AP; 1:1000), Tnni2 (Abcam, UK; ab184554; 1:1000), P21 (BOSTER, China; BM4382; 1:200), Ezh2 (Cell Signaling Technology, USA; 5246; 1:1000), Pcna (Servicebio, China; GB11010; 1:500), Ki67 (Abcam, UK; ab16667; 1:1000), β-actin (Santa Cruz Biotechnology, USA; sc-4777; 1:1000), Gapdh (BOSTER, China; BM3876; 1:200), Pax7 (Developmental Studies Hybridoma Bank; USA; 1:1000), eMyhc (Developmental Studies Hybridoma Bank, USA; BF-G6; 1:1000). The protein expression levels were normalized to corresponding β-actin or Gapdh and the western blotting bands signal intensities were quantified using ImageJ software.

### Immunofluorescence

C2C12 cells were cultured in 24-well plate and differentiated for 2–3 days. The immunofluorescence staining was performed according to the previous publication^44^. The antibodies and their dilutions were shown as following: Myog (Santa Cruz Biotechnology, USA; sc-12732; 1:50) and Myhc (Santa Cruz Biotechnology, USA; sc-376157; 1:200), a secondary antibody (anti-mouse CY3; Beyotime Biotechnology, China). The 4′, 6-diamidino-2-phenylindole (DAPI) was used to stain the cell nuclei. The images were visualized with a fluorescence microscope (IX51-A21PH, Olympus, Japan). The cell differentiation index was calculated by the ratio of the number of nuclei in the myotubes to the total number of nuclei in one field of view. For myoblast fusion, the cells were differentiated for 5 days. Then the cells were performed with myosin (Sigma, USA; M4276; 1:1000) immunofluorescence to test myoblast fusion. The cell fusion was calculated by the number of nuclei present in one myosin-positive cell indicated.

### Knockdown of *Neat1* in vivo by lentivirus infection

6-week C57 male mice were injected with 100 µL final volumes of lentivirus contained small interfere *Neat1* (LV3-sh*Neat1*) or control (LV3-shNC) at 2 × 10^7^ TU/ml into the right and left quadriceps (Qu), tibialis anterior (TA) and gastrocnemius (Gas) of the hind legs, respectively. LV3-sh*Neat1* or LV3-shNC was synthesized by GenePharma (China, Shanghai). LV3-sh*Neat1* or LV3-shNC was diluted in PBS and injected into Qu, TA and Gas of the hind legs every one week, after one month of injection, the mice were killed and the Qu, TA and Gas muscles of the hind legs were collected. For qPCR and western blotting analysis, the injection was performed in three mice. The Qu, TA and Gas muscles of the right or left hind legs of each injection mouse were collected, and used for total RNA and protein extraction. For myosin immunofluorescence, the Qu, TA and Gas muscles of the right or left hind legs of each injected mouse were fixed in 4% paraformaldehyde, respectively.

### Muscle injury and regeneration

For CTX injection, 6-week male mice were injected with 100 µL final volumes of LV3-sh*Neat1* or LV3-shNC at 2 × 10^7^ TU/ml into the right and left Gas muscles, respectively. One day after, the above mice were injected with 100ul of CTX at 80ug/ml into the Gas muscles at both hind legs. Mice were sacrificed and the Gas muscles were harvested at designed days, Mice were administered with 100 μg EdU (Thermo Fisher Scientific, Waltham, MA, USA) by intraperitoneal injection at 6 h before muscles harvesting. Extraction of the total RNAs and proteins were used for qPCR and western blotting analysis, respectively.

### Histology staining

For immunofluorescence staining of Pax7, EdU, Myog and eMyhc, the Gas muscles were harvested at day 3. Immunofluorescence staining on frozen muscle sections was performed in accordance with previous reports^21^, and images were visualized using a confocal laser scanning microscope (Zeiss, LSM800, Germany). The following dilutions were used for each antibody: Pax7 (Developmental Studies Hybridoma Bank; USA; 1:20), eMyhc (Developmental Studies Hybridoma Bank, USA; BF-G6; 1:100), Myog (Santa Cruz Biotechnology, USA; sc-12732; 1:20). To detect the EdU incorporation, the sections were performed using the Life Technologies Click-iT Kit according to the manufacturer’s instructions, and images were photographed using a confocal laser scanning microscope (LSM800; Zeiss). For H&E staining, the Gas muscles were harvested at day 0, 3, 7, and 15 after CTX injection. H&E of muscle sections was performed according to previous reported methods^21,45^, and the cross-section area of individual myofibers was visualized using Olympus DP80 upright Metallurgical Microscope (Olympus Corporation, Japan) and qualified using ImageJ software.

### RNA immunoprecipitation assay

RNA immunoprecipitation (RIP) assays were conducted using EZ-Magna RIP Kit (Millipore) according to the manufacturer’s instructions. Briefly, C2C12 cells were lysed with RIP lysis buffer, and incubated with 1 μg antibody (Ezh2, Abcam, ab3748; Suz12, Abcam, ab12073; IgG, Millipore) for RNA immunoprecipitation at 4 ℃ overnight. Then Protein A/G beads were added to the lysates to pull down antibody-protein-RNA complex. The detection of co-precipitated RNAs was performed by reverse-transcription polymerase chain reaction (RT-PCR).

### RNA pulldown assays

RNA pulldown was performed as previously described^46^. Briefly, linearizing DNA was biotin-labeled and in vitro transcribed using the Biotin RNA Labeling Mix and T7/SP6 RNA polymerase (Roche), and purified with the RNeasy Mini Kit (QIAGEN). One milligram of protein was incubated with 3 μg of biotinylated RNA for 1 h at room temperature. After that, 40ul Streptavidin-coupled Dynabeads (Invitrogen) were added to each reaction and incubated for 1 h at room temperature. Finally, the beads were washed in RIP buffer for five times, and the pull-downed proteins were used for western blotting. For mass spectrometry, the pull-downed proteins in C2C12 cells were separated by 10% SDS-PAGE, and then performed with silver staining. The differentially expressed bands were excised and analyzed by mass spectrometry (Novogene, Beijing, China).

### Chromatin immunoprecipitation assays

Chromatin immunoprecipitation (ChIP) assay was performed using ChIP Kit (Millipore, 17–371) according to the manufacturer’s instructions. Each ChIP reaction was performed using 1 μg of antibodies against Ezh2 (Abcam, ab3748), H3k27me3 (Abcam, ab6002) or IgG applied as negative control. Fold enrichment was quantified using qPCR. All promoter primers were listed in Supplementary Table S3.

### Chromatin Isolation by RNA Purification assays

Chromatin Isolation by RNA Purification (ChIRP) assay was performed using ChIRP Kit (Millipore#17–10495) according to the manufacturer’s instructions. Biotin-labeled *Neat1* probes were designed by Guangzhou Ribobio and divided into odd and even pools. C2C12 cells were cross-linked with 1% glutaraldehyde and lysed with lysis buffer before sonication for 4 h. Odd and even *Neat1* probes were added into samples for incubation at 37 °C for 4.5 h with rotating. The combined chromatin fragments were enriched by C-1 streptavidin beads and purified for qPCR experiment.

### Statistical analysis

All data were expressed as mean ± standard deviation (s.d.). Statistical analyses between different groups were performed using t-test. For all analyses, *p* < 0.05 was considered to be statistically significance.

## Results

### *Neat1* is up-regulated during myogenic differentiation and muscle regeneration

**Fig. 1:**
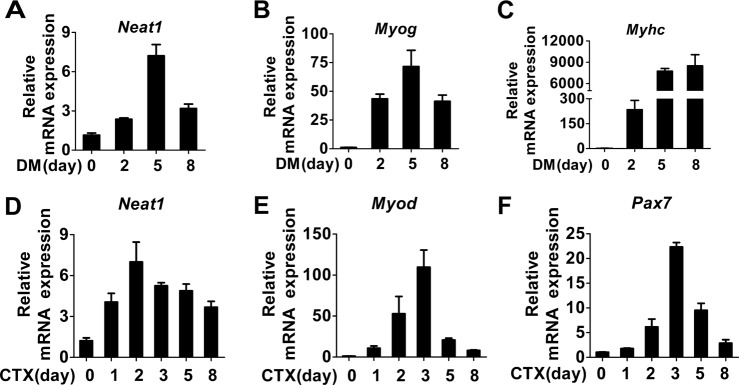
*Neat1* is upregulated during myogenesis and muscle regeneration. **a** qPCR results showing that *Neat1* expression was upregulated in C2C12 cells from day 0 (proliferating cells) to day 5 post differentiation but downregulated on day 8. **b**, **c** qPCR results showing that *Myog* expression was upregulated on days 0, 2, and 5 but downregulated on day 8 (**b**), whereas *Myhc* expression was upregulated during myoblast differentiation (**c**). **d–f** Hindlimb muscles were subjected to CTX injection and harvested at 0, 1, 2, 3, 5, and 8 days post-injury for RNA analysis. qPCR results showing that the expression of *Neat1* (**d**), *Myod* (**e**), and *Pax7* (**f**) genes was induced at the early stage of muscle regeneration but downregulated thereafter. Relative RNA levels were normalized to those of β*-*actin. All values represent the mean ± standard deviation (s.d.) of three independent experiments

To investigate the role of *Neat1* in myogenesis, we first examined the expression profiles of *Neat1* as well as *Myog* and *Myhc* during myogenic differentiation. The expression levels of *Neat1* and *Myog* were increased in C2C12 cells from day 0 (proliferating cells) to day 5 post differentiation but decreased on day 8, whereas *Myhc* expression gradually increased during differentiation (Fig. 1a–c). To further explore *Neat1* expression during muscle regeneration, we employed an extensively used muscle regeneration model in which intramuscular injection of cardiotoxin (CTX) leads to muscle injury and induces muscle regeneration^47^. The expression levels of *Neat1* along with *Pax7* and *Myod* were highly increased during the early stage of regeneration and then decreased when the newly formed fibers maturation and regeneration completed. We also found that *Neat1* expression peaked earlier than that of *Pax7* and *Myod* (Fig. 1d–f). These results suggest that *Neat1* is involved in myogenesis and muscle injury repair.

### *Neat1* promotes myoblast proliferation, but inhibits myogenic differentiation and fusion

**Fig. 2:**
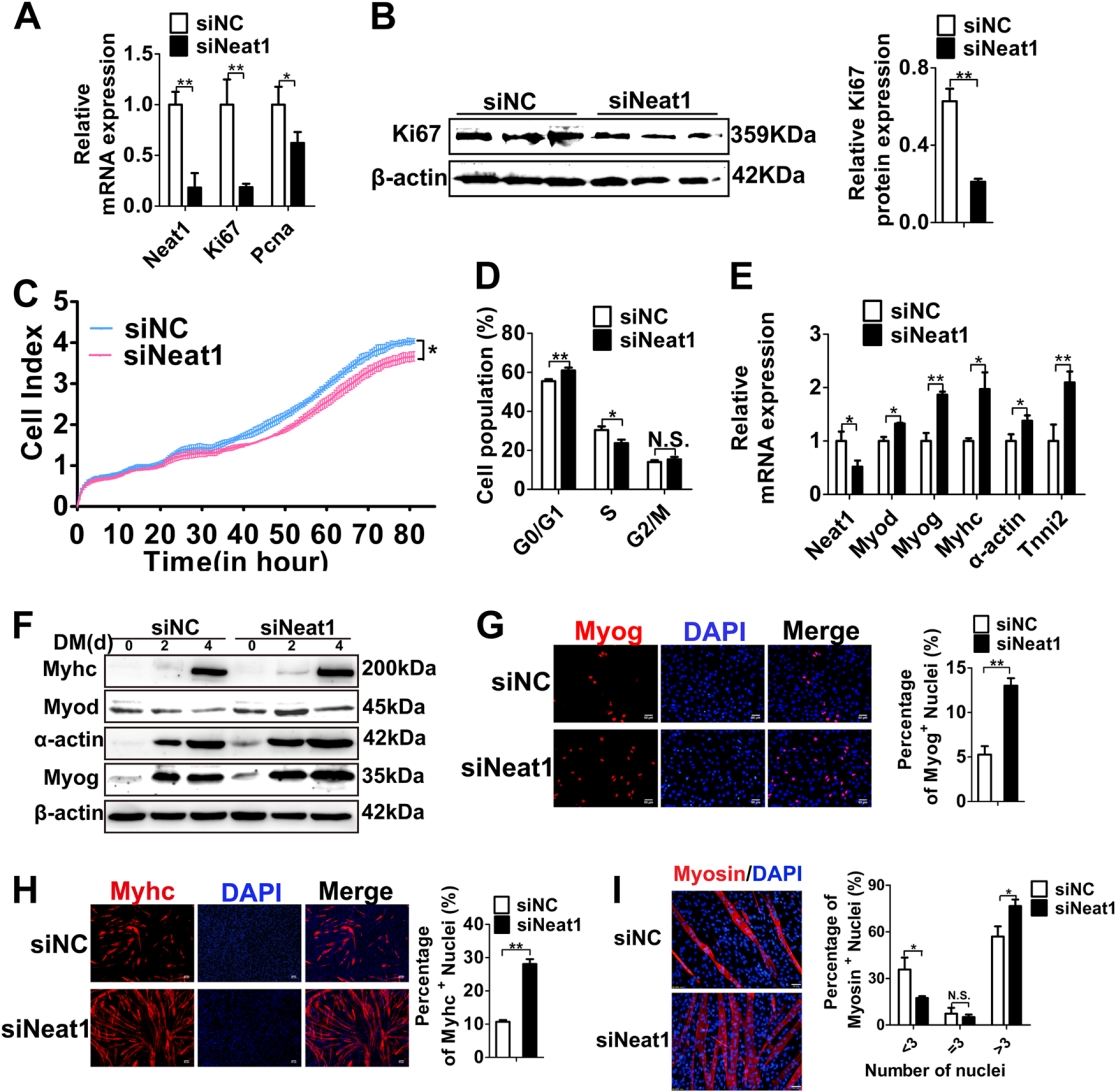
*Neat1* promotes the proliferation of C2C12 cells but inhibits myogenic differentiation and fusion. **a** qPCR results showing that the mRNA expression of *Ki67* and *Pcna* was significantly decreased by *Neat1* knockdown. **b** Western blotting analysis showing that Ki67 protein expression was significantly decreased by *Neat1* knockdown. **c** The RTCA xCELLigence assay demonstrating that cell growth dynamics were significantly reduced by *Neat1* knockdown. **d** The quantification of EdU-PI flow cytometry results showing that the proportion of cells in S phase was significantly reduced by *Neat1* knockdown. **e** qPCR results showing that the mRNA expression of *Myod*, *Myog*, *Myhc*, α-actin, and *Tnni2* was significantly increased by *Neat1* knockdown in C2C12 cells on day 2 post differentiation. **f** Western blotting analysis showing that the protein expression of Myod, Myog, Myhc, and α-actin was significantly increased by *Neat1* knockdown in C2C12 cells on days 0, 2, and 4 post differentiation. **g** Immunofluorescence staining of Myog showing that Myog protein expression was significantly increased by *Neat1* knockdown on day 2 post-transfection. Cell nuclei were stained with DAPI. The number of Myog^+^ cells was quantified using ImageJ software. **h** Immunofluorescence staining of Myhc showing significant upregulation of C2C12 differentiation by *Neat1* knockdown on day 3 post-transfection. The number of Myhc^+^ cells was quantified using ImageJ software. **i** Immunofluorescence staining of myosin in C2C12 cells differentiated for 5 days showing that knockdown of *Neat1* significant enhanced the myoblast fusion. Relative RNA and protein levels were normalized to those of β-actin. All values represent the mean ± s.d. of three independent experiments. *p < 0.05, **p < 0.01, N.S. indicates not significant

*NEAT1* promotes tumor growth in many cancer cells, including prostate cancer^37^, breast cancer cell lines^38^, colorectal cancer^39^. To investigate the roles of *Neat1* in myoblast proliferation, we conducted *Neat1* knockdown and overexpression experiments in C2C12 cells. *Neat1* knockdown led to a significant reduction in *Ki67* and *Pcna* mRNA expression and Ki67 protein expression (Fig. 2a, b), whereas *Neat1* overexpression had the opposite effect (Supplementary Fig. 1a–b). RTCA xCELLigence, EdU-PI flow cytometry assays confirmed the effects of *Neat1* on cell proliferation. The RTCA xCELLigence assay suggested that *Neat1* knockdown significantly reduced cell growth (Fig. 2c), while *Neat1* overexpression enhanced cell growth (Supplementary Fig. 1c). EdU-PI flow cytometry assays showed a significant decrease and increase in DNA replication (S-phase) after *Neat1* knockdown and overexpression, respectively (Fig. 2d and Supplementary Fig. 1d-e). These observations indicate that *Neat1* promotes the proliferation of C2C12 cells.

To verify the functions of *Neat1* during C2C12 differentiation, *Neat1* was knocked down and overexpressed during C2C12 cell differentiation. Knockdown of *Neat1* increased the mRNA expression of myogenic marker genes, such as *Myod*, *Myog*, *Myhc*, α-actin, and *Tnni2* (Fig. 2e). *Neat1* knockdown also enhanced myogenic differentiation, as indicated by a significant increase in the protein expression of these genes during cell differentiation (Fig. 2f and Supplementary Fig. 2a). Immunofluorescence staining of Myog and Myhc revealed an increased number of Myog^+^ and Myhc^+^ cells (Fig. 2g, h). These observations were also confirmed by *Neat1* overexpression (Supplementary Fig. 2b-f). Together, these data demonstrate that *Neat1* inhibits myoblast differentiation.

Finally, to investigate whether *Neat1* affects myoblasts fusion, myosin immunofluorescence staining was used to analyze myoblasts fusion after *Neat1* knockdown or overexpression. The results showed that *Neat1* knockdown increased the ratio of myotubes with more than three nuclei, while *Neat1* overexpression decreased the ratio of myotubes with more than three nuclei (Fig. 2i and Supplementary Fig. 2g). In addition, *Neat1* knockdown and overexpression significantly increased and decreased the fusion marker gene *Myomaker* expression, respectively (Supplementary Fig. 2h–i), suggesting *Neat1* inhibits myoblasts fusion.

### Knockdown of *Neat1* promotes postnatal muscle growth in vivo

**Fig. 3:**
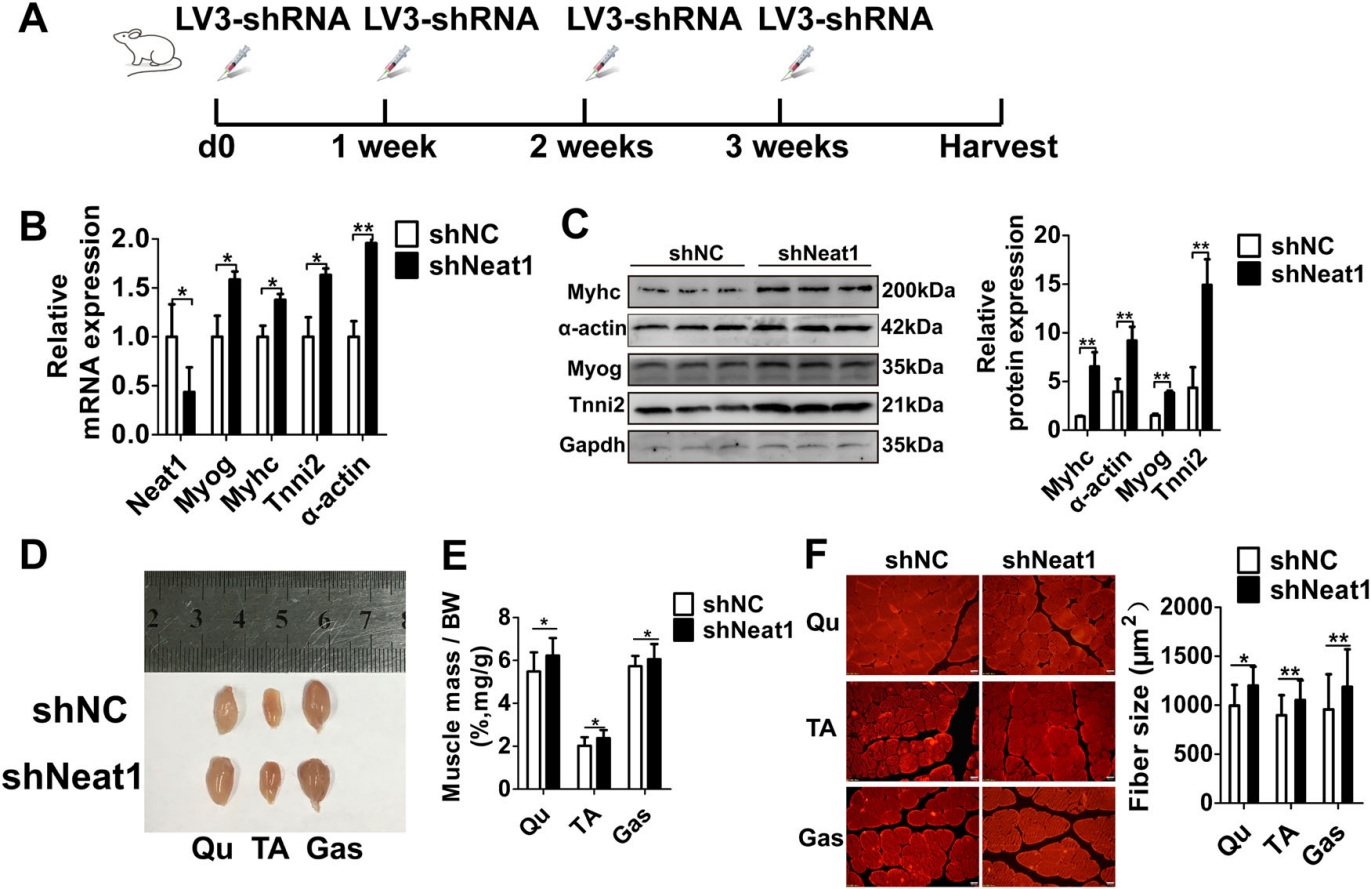
Knockdown of *Neat1* improves muscle growth in vivo. **a** The injection scheme for LV3-sh*Neat1* or LV3-shNC particles into the right or left hindlimb muscles of C57 mice. The injection was given every one week for one month. **b** qPCR results showing that *Neat1* expression was reduced after LV3-sh*Neat1* particle injection, while the mRNA expression of *Myog*, *Myhc*, *α-actin*, and *Tnni2* was significantly increased after LV3-sh*Neat1* particle injection. **c** Western blotting analysis showing that the protein expression of Myog, Myhc, α-actin, and Tnni2 was increased after LV3-sh*Neat1* particle injection. The protein levels of these genes were quantified using ImageJ software. **d** Representative photograph of the three muscles from the right or left hindlimbs showing that the volume of the Qu, TA, and Gas muscles of the right hindlimb were larger than those of the left hindlimb. **e** Quantification of the weight of three muscles from the right or left hindlimbs of 12 injected mice showing that the weight of the Qu, TA, and Gas muscles of the right hindlimb were higher than those of the left hindlimb. **f** Representative photograph of myosin immunofluorescence staining in Qu, TA, and Gas muscles from the right or left hindlimb following injection with LV3-sh*Neat1* or LV3-shNC particles. Compared with LV3-shNC particle injection, LV3-sh*Neat1* particle injection increased the average cross-sectional areas of the indicated muscles. The fiber sizes of the Qu, TA, and Gas muscles were quantified using ImageJ. Relative RNA and protein levels were normalized to those of Gapdh. All values represent the mean ± s.d. of three independent experiments. *p < 0.05, **p < 0.01

To investigate the effects of *Neat1* on muscle growth in vivo, 6-week-old C57 mice were injected with LV3-sh*Neat1* or LV3-shNC particles into the right and left hindlimbs, respectively. The injection scheme is shown in Fig. 3a. The injection of LV3-sh*Neat1* particles led to a significant reduction of *Neat1* expression (Fig. 3b). The mRNA and protein levels of *Myog*, *Myhc*, *Tnni2*, and *α-actin* genes were significantly increased after *Neat1* knockdown (Fig. 3b, c). Further, the volume and weight of the Qu, TA, and Gas muscles in *Neat1* knockdown groups were larger than those of the controls (Fig. 3d, e). Immunofluorescence staining for myosin showed that the cross-sectional areas of the Qu, TA, and Gas muscles injected with LV3-sh*Neat1* particles were dramatically larger than those injected with LV3-shNC particles (Fig. 3f). These results indicate that *Neat1* knockdown enhances muscle growth.

### *Neat1* knockdown delays muscle regeneration after CTX injection in vivo

**Fig. 4:**
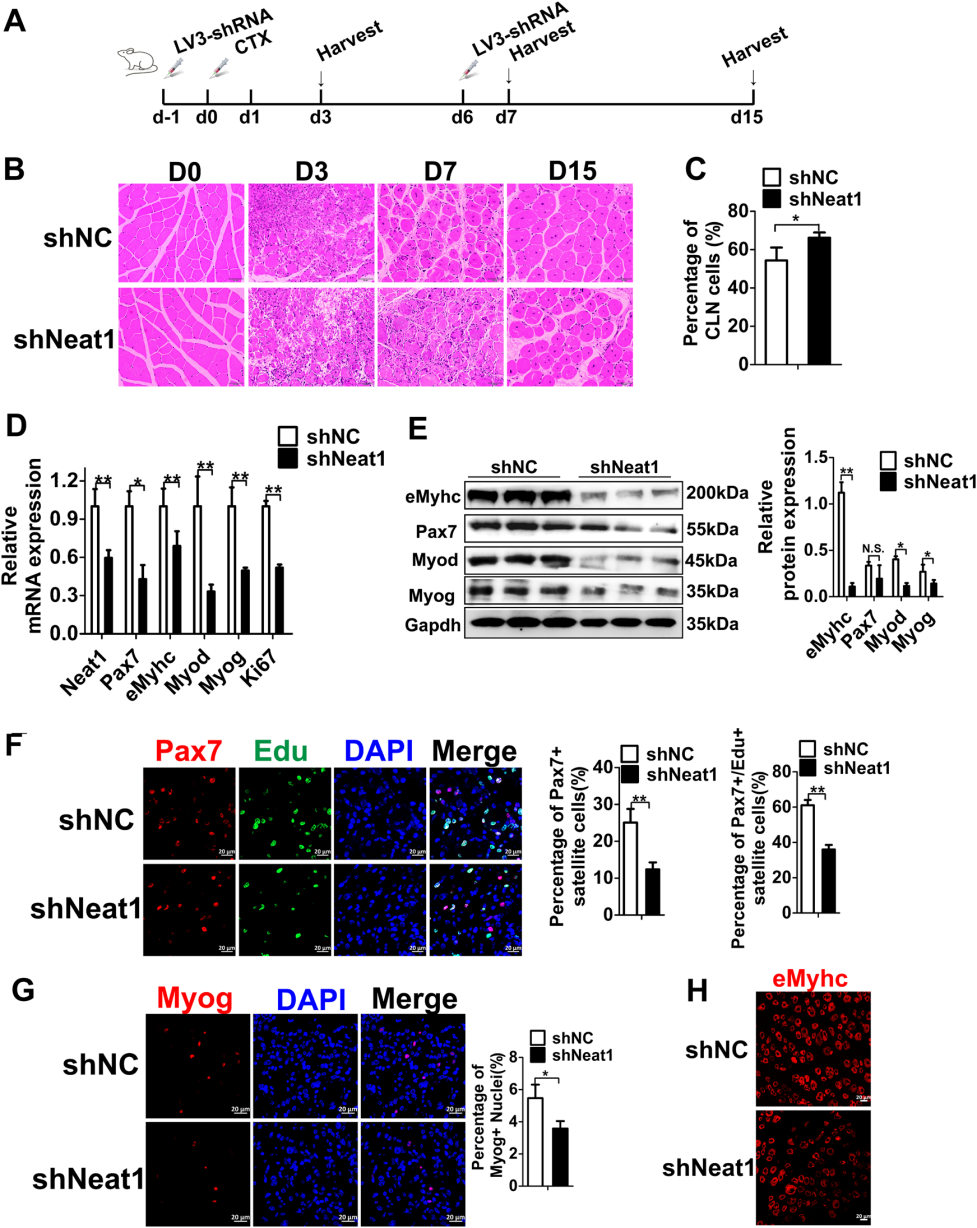
*Neat1* inhibition delays muscle regeneration following CTX injection in vivo. **a** The scheme for LV3-sh*Neat1* or LV3-shNC particle injection and CTX injection into the Gas muscles and harvesting time points for subsequent analysis. **b** H&E staining results showing that LV3-sh*Neat1* particle injection significantly delayed muscle regeneration compared with LV3-shNC particle injection. The injected muscles were harvested on days 0, 3, 7, and 15 post-CTX injections and used for H&E staining. **c** The quantification of the percentage of fibers with central localized nuclei (CLN) at 15 days after CTX injection. The results showed that the percentage of fibers with CLN were significantly higher in LV3-sh*Neat1* groups than that in LV3-shNC groups. **d** qPCR results showing that the mRNA expression levels of *Neat1*, *Pax7*, *Myod*, *Myog*, *eMyhc*, and *Ki67* genes were remarkably reduced after LV3-sh*Neat1* particle injection. The injected muscles were harvested on day 7 post-CTX injection. **e** Western blotting analysis showing that the protein expression levels of Pax7, Myod, Myog, and eMyhc were reduced after LV3-sh*Neat1* particle injection. The protein levels of these genes were quantified using ImageJ software. The injected muscles were harvested on day 7 post-CTX injection. **f** Immunofluorescence staining of Pax7, EdU and DAPI in muscle sections on day 3 post-CTX injection showing that Pax7^+^ SCs and proliferating SCs were reduced after LV3-sh*Neat1* particle injection. The percentage of Pax7^+^ SCs were indicated for the proportion of the number of Pax7^+^ nuclei in total number of DAPI, the percentage of proliferating satellite cells were indicated for the proportion of the number of Pax7^+^/EdU^+^ nuclei in total number of Pax7^+^ nuclei. **g**, **h** Immunofluorescence staining of Myog (**g**) and eMyhc (**h**) in muscle sections on day 3 post-CTX showing significantly reduced expression of these proteins after LV3-sh*Neat1* particle injection. Relative RNA and protein levels were normalized to those of Gapdh. All values represent the mean ± s.d. of three independent experiments. *p < 0.05, **p < 0.01, N.S. indicates not significant

To determine whether *Neat1* regulates CTX-induced muscle regeneration, 6-week-old C57 mice were injected with LV3-sh*Neat1* or LV3-shNC particles into the Gas muscles of the right and left hindlimbs, respectively, followed by CTX injection (Fig. 4a). The muscle regeneration phenotype was evaluated by H&E staining of muscle sections on days 0, 3, 7, and 15 after CTX treatment. On day 7 post-CTX treatment, the LV3-sh*Neat1* injection group displayed more inflammatory cells and fewer newly formed myofibers. On day 15 post-CTX treatment, the newly formed myofibers with the central nucleus were smaller in the LV3-sh*Neat1* injection group than those of the LV3-shNC injection group (Fig. 4b), and the percentage of fibers with the central nucleus was higher in LV3-sh*Neat1* group than that in LV3-shNC groups (Fig. 4c**)**. Knockdown of *Neat1* also led to a decrease in both the mRNA and protein levels of *Pax7*, *Myod*, *Myog*, and *eMyhc* genes, as well as the *Ki67* mRNA level, on day 7 post-CTX injection (Fig. 4d, e). Immunofluorescence staining in muscle sections showed that the percentage of both Pax7^+^ SCs and proliferating Pax7 (Pax7^+^/EdU^+^) SCs were reduced in the LV3-sh*Neat1* injection group compared with the controls on day 3 post-CTX injection (Fig. 4f). Immunofluorescence staining in muscle sections also showed that knockdown of *Neat1* decreased the number of cells with positive staining of Myog and eMyhc on day 3 post-CTX injection (Fig. 4g, h). Reduced Myog and eMyhc expression in the LV3-sh*Neat1* injection groups is likely the consequence of impaired SC proliferation rather than impaired differentiation, as *Neat1* knockdown promoted Myog and Myhc expression. Together, these results suggest that *Neat1* knockdown delays muscle regeneration following CTX treatment.

### *Neat1* physically interacts with Ezh2

**Fig. 5:**
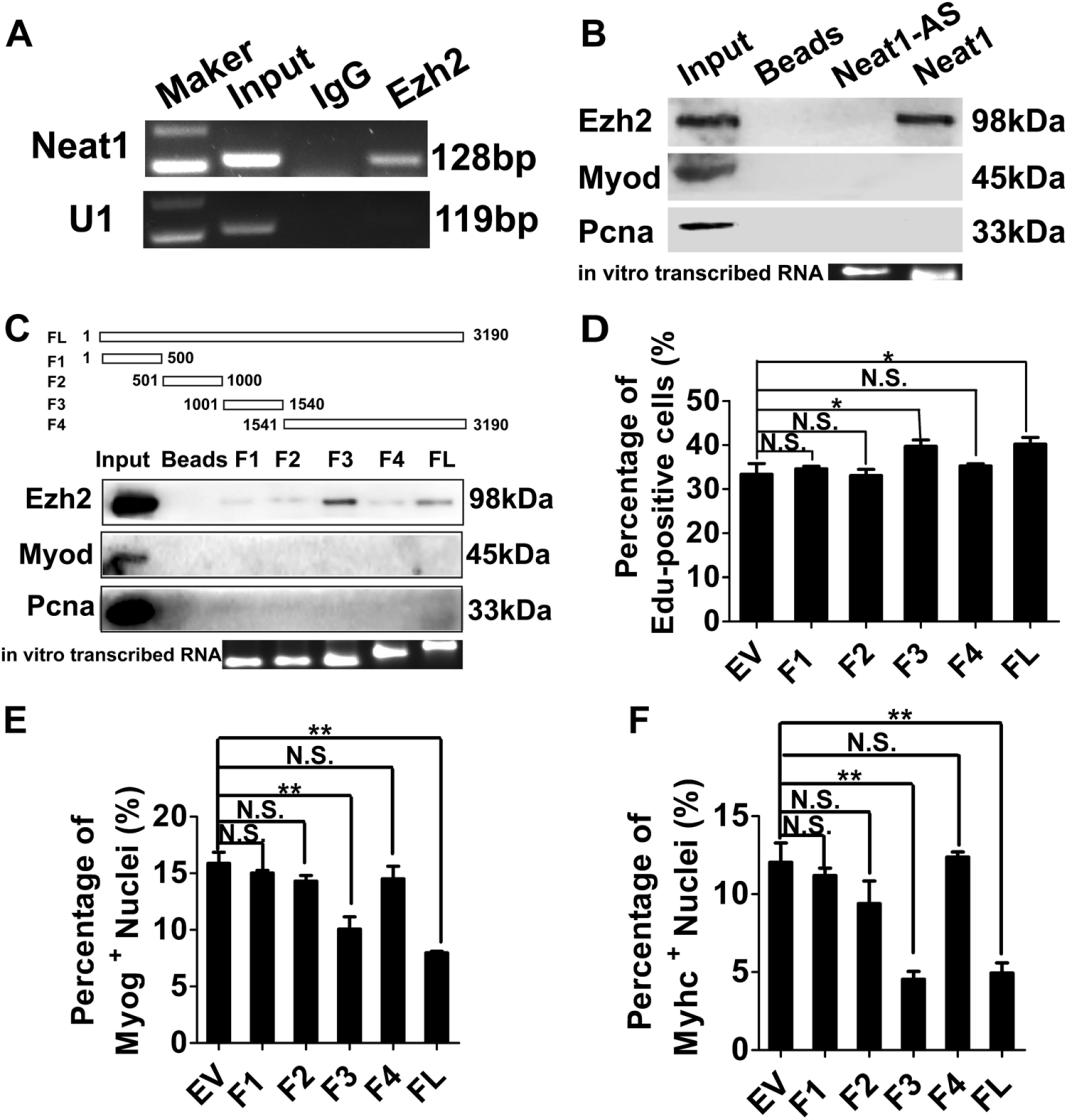
*Neat1* interacts with Ezh2. **a** RNA immunoprecipitation (RIP) assays performed in C2C12 cells using Ezh2 antibodies. The retrieved *Neat1* transcripts were assessed by PCR. **b** Biotin-labeled full-length *Neat1* was used to pull down Ezh2. Western blotting analysis was performed to detect the precipitated Ezh2 protein. Myod and Pcna were used as negative controls. **c** The interactions between a series of *Neat1* fragments (FL, F1, F2, F3, F4) and Ezh2 assessed by RNA pull-down assays. **d** Transfection of a series of *Neat1* fragments (FL, F1, F2, F3, and F4) into C2C12 cells, followed by EdU staining in the cells on day 2 after transfection. The quantification of EdU staining results showed that the overexpression of F3, but not the other fragments, and full-length *Neat1* significantly promoted C2C12 cell proliferation. **e**, **f** Transfection of a series of *Neat1* fragments (FL, F1, F2, F3, and F4) into C2C12 cells, followed by immunofluorescence staining of Myog and Myhc at 2 days after differentiation. The quantification of immunofluorescence staining results showed that the overexpression of F3, but not the other fragments, and full-length *Neat1* significantly inhibited Myog protein expression (**e**) and C2C12 cell differentiation (**f**). U1 was used as controls for the RIP assays. Myod and Pcna were used as controls for RNA pull-down assays. All values represent the mean ± s.d. of three independent experiments. *p < 0.05, **p < 0.01, N.S. indicates not significant

As a well-known nuclear lncRNA, *Neat1* may play a role at the transcriptional level. A recent study revealed that *NEAT1* interacts with EZH2 in human glioblastoma cells^48^. Our previous studies showed that Ezh2 plays important roles in myoblast proliferation and differentiation by increasing the level of H3k27me3 binding at gene promoters^27^; therefore, we inferred that *Neat1* may also regulate myoblast proliferation and myogenic differentiation by interacting with Ezh2. First, we performed RNA immunoprecipitation (RIP) assays to confirm the interaction between *Neat1* and Ezh2, and as expected, *Neat1* was significantly enriched on the Ezh2 antibody compared with the IgG antibody (negative control) (Fig. 5a). To confirm the interaction, biotin-labeled full-length *Neat1* was used to pull down target proteins. RNA pull-down assays revealed that *Neat1* transcripts pulled down endogenous Ezh2 but not Myod or Pcna (Fig. 5b), and there was no mutual effect between *Neat1* and *Ezh2* expression (Supplementary Fig. 3a–c), suggesting that *Neat1* physically binds to Ezh2. As PRC2 contains several members, such as Ezh2 and Suz12, we also evaluated the interaction between *Neat1* and Suz12. As expected, *Neat1* was also pulled down by the Suz12 antibody (Supplementary Fig. 3d). Next, a series of deletions in full-length *Neat1* was performed to determine the core binding domain (Fig. 5c). Interestingly, the third fragment (1001–1540, F3) was efficiently pulled down Ezh2, whereas fragments F1, F2, and F4 rarely pulled down Ezh2 (Fig. 5c). To further verify the core functional domain of *Neat1*, F1, F2, F3, F4, and full-length *Neat1* were overexpressed in C2C12 cells. EdU staining showed that the overexpression of F3 and full-length *Neat1* significantly increased EdU incorporation compared with the control (Fig. 5d and Supplementary Fig. 3e). Immunofluorescence staining also revealed a significantly decreased number of Myog^+^ and Myhc^+^ cells when F3 and full-length *Neat1* were overexpressed (Fig. 5e, f and Supplementary Fig. 3f–g). These results indicate that the F3 fragment of *Neat1* is required for its recruitment of Ezh2 in myogenesis. Besides, we also identified 122 *Neat1*-binding proteins in C2C12 cells by mass spectrometry including some known interacting paraspecle proteins (Sfpq, Hnrnpr, Hnrnpm and Matr3) and some proteins involved in muscle development and disease (Eef2, Dnm2, Trim28, Trpv2, Wfs1, Pml, Vcp, Lmna and Matr3) (Supplementary Fig. 3h and Supplementary Table 4).

### *Neat1* enhances the proliferation of C2C12 cells via Ezh2-mediated H3k27me3 enrichment at the *P21* promoter

**Fig. 6:**
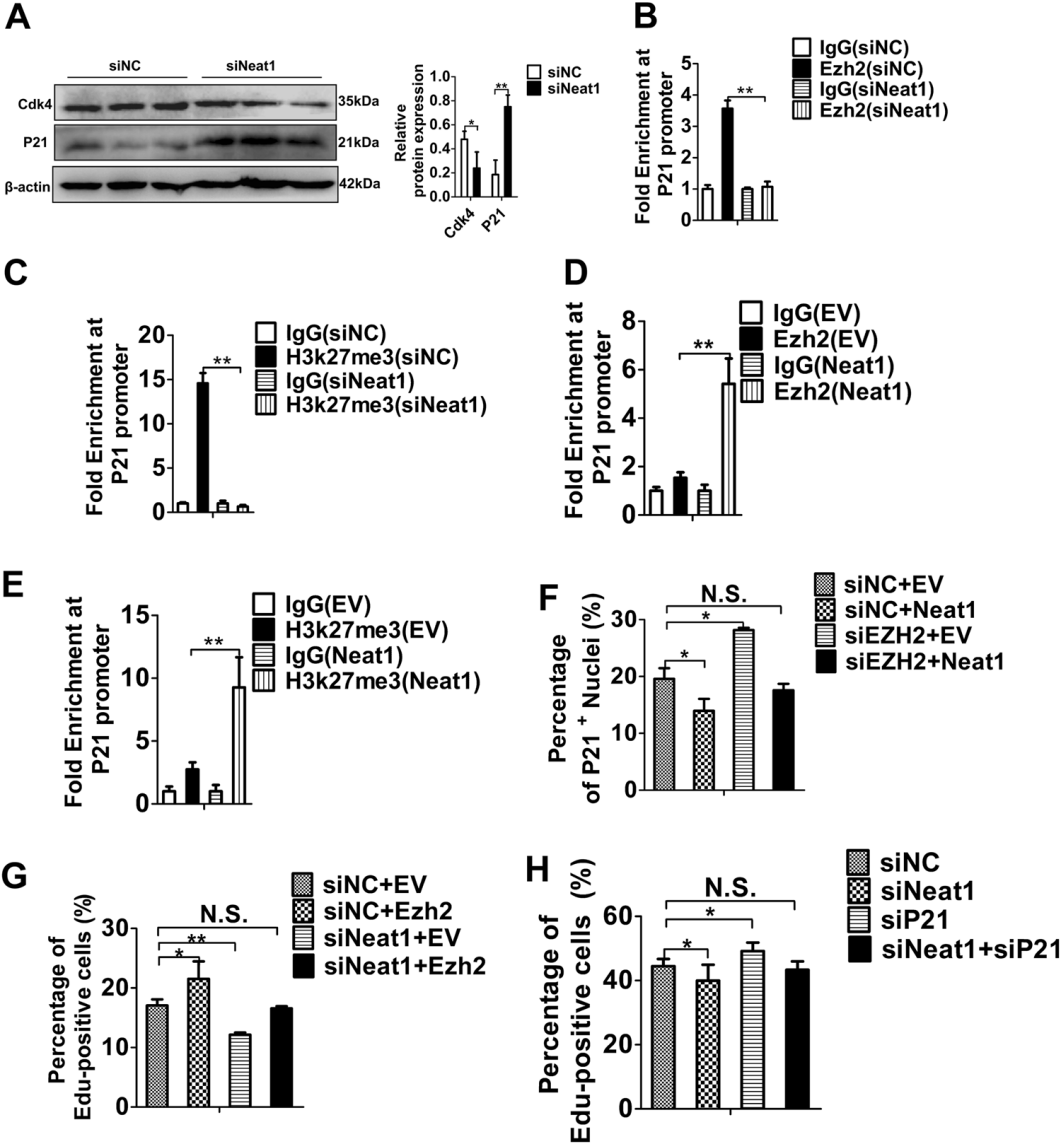
*Neat1* inhibits *P21* expression throngh Ezh2. **a** Western blotting analysis showing that *Neat1* knockdown enhanced P21 protein expression but decreased Cdk4 protein expression in C2C12 cells. The relative protein levels of P21 and Cdk4 were quantified using ImageJ software. **b**, **c** ChIP-qPCR results revealed that the enrichments of Ezh2 (**b**) and H3k27me3 (**c**) at the *P21* promoter were significantly decreased after *Neat1* knockdown. **d**, **e** ChIP-qPCR results revealed that the enrichments of Ezh2 (**d**) and H3k27me3 (**e**) at the *P21* promoter were significantly increased after *Neat1* overexpression. **f** Co-transfection of *Ezh2* siRNA fragment and *Neat1* expression vector in C2C12 cells for 2 days. Immunofluorescence staining of P21 was performed, and P21 expression was quantified by ImageJ. The quantification of P21 immunofluorescence staining results showed that the overexpression of *Neat1* inhibited P21 protein expression, but had no significant effect on P21 expression after co-transfection with *Ezh2* siRNA fragment. **g** *Neat1* siRNA fragment and *Ezh2* expression vector were co-transfected into C2C12 cells and the cells were performed with EdU staining at 2 days after transfection. The percentage of EdU^+^ cells was quantified. The quantification of EdU staining results showed that *Neat1* knockdown inhibited myoblast proliferation. After co-transfected with *Ezh2* expression vector, *Neat1* knockdown can not inhibit myoblast proliferation. **h** *Neat1* and *P21* siRNA fragments were co-transfected into C2C12 cells and the cells were performed with EdU staining at 2 days after transfection. The quantification of EdU staining results showed that *Neat1* knockdown significantly reduced the percentage of EdU^+^ cells, but did not reduce the number of EdU^+^ cells after co-transfection with *P21* siRNA fragment. Protein levels were normalized to those of β-actin. All values represent the mean ± s.d. of three independent experiments. *p < 0.05, **p < 0.01, N.S. indicates not significant

Previous studies have shown that lncRNAs such as *SNHG20*^49^, *Xist*^50^ and *SYSIL*^27^ repress the cyclin-dependent kinase inhibitor 1 A (*P21*) by interacting with Ezh2, as well as promote cell proliferation. Our above mentioned results showed that *Neat1* promotes proliferation and interacts with Ezh2 in C2C12 cells (Fig. 2a–d and Supplementary Fig. 1 and Fig. 5). Further, the overexpression of *P21* significantly reduced the number of EdU^+^ cells, whereas *Ezh2* overexpression increased the number of EdU^+^ cells (Supplementary Fig. 4a-b), demonstrating that *P21* and *Ezh2* have the opposite effects on C2C12 cell proliferation. Thus, we inferred that *Neat1* facilitates C2C12 cell proliferation by Ezh2-mediated inhibition of *P21* expression. First, we explored whether *Neat1* regulates *P21* expression and found that knockdown of *Neat1* inhibited cell cycle-dependent kinase 4 (Cdk4) and enhanced P21 protein expression, indicating that *Neat1* inhibits *P21* expression in C2C12 cells (Fig. 6a). A previous study showed that Ezh2 inhibits *P21* expression by directly binding to its promoter^27^. Therefore, we performed ChIP-qPCR assays to explore whether *Neat1* affects the Ezh2- and H3k27me3-binding capacities at the *P21* promoter. Knockdown and overexpression of *Neat1* decreased and increased the enrichment of Ezh2 and H3k27me3 at the *P21* promoter, respectively (Fig. 6b–e). To further elucidate whether *Neat1* affected *P21* expression through Ezh2, *Ezh2* siRNA fragment and *Neat1* expression vector were co-transfected into C2C12 cells. Immunofluorescence staining of P21 revealed that *Neat1* overexpression inhibited P21 expression, but had no effect when co-transfected with *Ezh2* siRNA fragments (Fig. 6f and Supplementary Fig. 4c). We also co-transfected *Neat1* siRNA fragments with *Ezh2* expression vector in C2C12 cells and assessed cell proliferation by EdU staining. Knockdown of *Neat1* significantly reduced the percentage of EdU^+^ cells. After transfection with *Ezh2* expression vector, *Neat1* knockdown did not reduce the number of EdU^+^ cells (Fig. 6g and Supplementary Fig. 4d), indicating that *Neat1* regulation of myoblast proliferation is dependent on Ezh2. To further confirm whether *Neat1* promoted cell proliferation through *P21* pathway, *Neat1* and *P21* siRNA fragments were co-transfected into C2C12 cells, and then the cell proliferation ability was detected by EdU staining. The results showed that *Neat1* knockdown significantly reduced the percentage of EdU^+^ cells, but had no effect after co-transfection with *P21* siRNA fragment **(**Fig. 6h and Supplementary Fig. 4e). Together, these results suggest that *Neat1* inhibits *P21* expression by increasing the Ezh2-binding capacities at its promoter, thereby promoting C2C12 cell proliferation.

### *Neat1* inhibits myogenic differentiation by epigenetically silencing the expression of myogenic markers

**Fig. 7:**
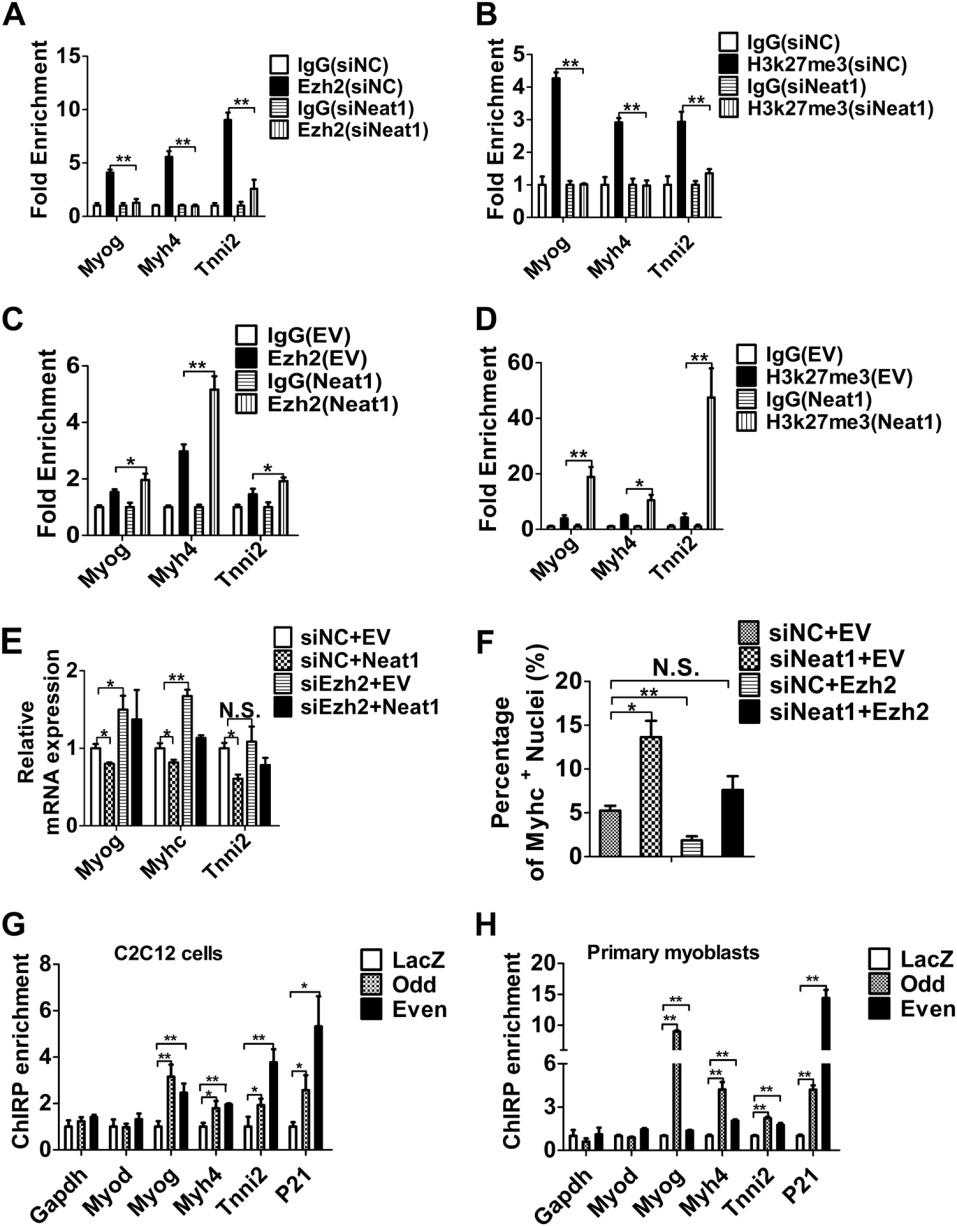
*Neat1* inhibits the expression of myogenic genes via Ezh2. **a**, **b** ChIP-qPCR results revealing that *Neat1* knockdown significantly decreased the enrichments of Ezh2 (**a**) and H3k27me3 (**b**) at the *Myog*, *Myh4*, and *Tnni2* promoters. **c**, **d** ChIP-qPCR results indicating *Neat1* overexpression significantly increased the enrichments of Ezh2 (**c**) and H3k27me3 (**d**) at the *Myog*, *Myh4*, and *Tnni2* promoters. **e** *Neat1* expression vector and *Ezh2* siRNA fragment were co-transfected into C2C12 cells. The indicated genes expression were measured by qPCR after *Neat1* expression vector and *Ezh2* siRNA fragment were co-transfected 3 days post differentiation. The results showed that overexpression of *Neat1* inhibits the expression of *Myog*, *Myhc* and *Tnni2*, but had no significant effect when co-transfected with *Ezh2* siRNA fragment. **f** *Neat1* siRNA fragment was con-transfected with *Ezh2* expression vector into C2C12 cells at 5 days post differentiation, the cells were performed with Myhc immunofluorescence staining. The quantification of Myhc immunofluorescence staining results showed *Neat1* knockdown alone increased Myhc protein expression but not when co-transfected with *Ezh2* expression vector. **g**, **h** ChIRP-qPCR results revealed that *Neat1* binds directly to the *Myog*, *Myh4*, and *Tnni2* promoters but not to the *Myod* promoter in C2C12 cells (**g**) and primary myoblasts (**h**). Relative RNA levels were normalized to those of β-actin. All values represent the mean ± s.d. of three independent experiments. *p < 0.05, **p < 0.01, N.S. indicates not significant

Previous studies have shown that Ezh2 suppresses myogenic differentiation by increasing levels of the epigenetic silencing marker H3k27me3 to repress the transcription of myogenic markers such as *Myog*^18,51^, *myh4*^18,51,52^, and *Tnni2*^52^. Therefore, we performed ChIP assays to determine whether *Neat1* affects the inhibitory effects of Ezh2 on the expression of these myogenic genes. ChIP-qPCR assays suggested that knockdown of *Neat1* decreased the binding of Ezh2 and H3k27me3 at the *Myog*, *Myh4*, and *Tnni2* gene promoters (Fig. 7a, b), which was confirmed by *Neat1* overexpression (Fig. 7c, d). To further elucidate whether *Neat1* affects these target genes via Ezh2, *Neat1* expression vector and *Ezh2* siRNA fragment were co-transfected into C2C12 cells. As expected, the overexpression of *Neat1* remarkably reduced the expression of *Myog*, *Myhc*, and *Tnni2*, but not when co-transfected with the *Ezh2* siRNA fragments (Fig. 7e and Supplementary Fig. 5a–b). Immunofluorescence staining of Myhc also showed that *Neat1* knockdown enhanced Myhc expression, but not when co-transfected with *Ezh2* expression vector (Fig. 7f and Supplementary Fig. 5c).

Lastly, we performed chromatin isolation by RNA purification (ChIRP) to confirm that *Neat1* binds directly to the *Myog*, *Myh4*, *Tnni2*, and *P21* gene promoters but not to the *Myod* promoter in C2C12 cells and mouse primary myoblasts (Fig. 7g, h), consistent with the pattern of Ezh2 occupancy at its target genes (Fig. 6b–d and Fig. 7a, c and Supplementary Fig. 5d), indicating *Neat1* regulated *Myod* expression independent of Ezh2. Altogether, these results suggest that *Neat1* suppresses C2C12 myogenic differentiation mainly by increasing Ezh2 enrichment at the promoters of target genes.

## Discussion

*NEAT1* is involved in multiple biological processes in vitro and in vivo. In vitro, *NEAT1* affects the proliferation, migration, invasion, and apoptosis of multiple cancer cells^53^; for example, *NEAT1* promotes the proliferation and invasion of colorectal cancer cells^54^. Knockdown of *NEAT1* suppresses the migration and invasion of glioma cells^55^. *NEAT1* is regulated by c-myc and inhibits imatinib-induced apoptosis of chronic myeloid leukemia cells^56^. Besides, *NEAT1* also plays an important role in vascular smooth muscle cell phenotypic switching^36^. In vivo, *NEAT1* is overexpressed in many solid tumors, including small cell lung cancer^57^ and hepatocellular carcinoma^58,59^. *Neat1* knockout mice display impaired corpus luteum differentiation^60^. The genetic ablation of *Neat1* leads to abnormal mammary gland morphogenesis and lactation defects^61^. *Neat1* exerts anti-apoptotic and anti-inflammatory functions in C57BL/6 mice after traumatic brain injury^62^. The functions of *Neat1* in myogenesis and skeletal muscle development remain unexplored. Here, we demonstrated that *Neat1* promotes myoblast proliferation and inhibits myogenic differentiation. Moreover, knockdown of *Neat1* improved the cross-sectional area of muscle fibers, mainly by increasing the expression of myogenic genes, and delaying muscle regeneration, primarily via a reduction in the number of Pax7^+^ cells. In general, our study found a previously unidentified function of *Neat1* in regulating muscle development and regeneration.

LncRNAs regulate gene expression at the transcriptional and post-transcriptional levels or by chromatin modifications^63–65^. As a well-known nuclear lncRNA, *NEAT1* functions mainly as a transcriptional regulator. Capture hybridization analysis of RNA targets (CHART) analysis revealed that *NEAT1* binds directly to both the transcriptional start sites and transcriptional termination sites of target genes. CHART-mass spectrometry assays identified a large number of *NEAT1*-interacting proteins^66^, suggesting that the function of *NEAT1* in transcriptional regulation may be mediated by many proteins. In the present study, we found that *Neat1* promoted myoblast proliferation and inhibited myogenic differentiation by guiding Ezh2 to target gene promoters, such as *Myog*, *Myh4*, *Tnni2*, and *P21*, and repressed their transcription. Ezh2 is an important epigenetic inhibitory factor involved in many biological processes, including myogenesis. Ezh2 suppresses myogenic differentiation mainly by inhibiting the expression of myogenic marker genes, such as *Myog*, *myh4*, and *Mck*^18,51,52^. A previous study showed that the conditional knockout of Ezh2 in SCs resulted in decreased muscle regeneration and number of Pax7^+^ cells^67^, consistent with the phenotype of *Neat1* knockdown. Moreover, *Neat1* knockdown in vivo increased the expression of myogenic genes and the myofiber cross-sectional area, which may be also mediated by Ezh2, because Ezh2 muscle-specific knockout SCs also enhanced myogenic differentiation reflected by increased *Myog* expression^67^. Therefore, we conclude that the *Neat1* is an important regulator of Ezh2-mediated epigenetic regulation in myogenesis and muscle regeneration. In addition to interacting with Ezh2, *Neat1* may regulate myogenesis via other proteins or signaling pathways, because *Neat1* also inhibited *Myod* expression in an Ezh2-independent manner. Further endeavors will be devoted to determining the other mechanisms by which *Neat1* affects myogenesis.

LncRNAs are highly conserved in terms of their position within the genome, and conserved lncRNAs may play similar functions among species. For example, *linc-YY1* and *lncMyoD* are both conserved in their genomic positions and are involved in myogenesis in both mouse and human^68,69^. Compared with protein-coding genes, lncRNAs have low sequence conservation. However, some lncRNAs that possess “ultraconserved” regions also have conserved functions^70,71^. For example, the lncRNA *THOR* exerts a carcinogenic role by interacting with IGF2BP1 via an ultraconserved region in human, mouse, and zebrafish^72^. In the current study, we observed that the F3 region (1001–1540 bp) of *Neat1* encompasses the functional domain due to its interaction with Ezh2 and regulation of myogenesis, and further sequence alignment analysis demonstrated that this 1001–1540 bp region is also conserved between mouse and human (Supplementary Fig. 6), indicating a potentially ultraconserved region. In addition, a previous study showed that lncRNA higher-order structures are highly conserved, and these conserved secondary and tertiary structures are related to their biological functions and protein-binding potential^73,74^. Therefore, we speculated that this 1001–1540 bp region of *Neat1* may also contribute to the formation of *Neat1* higher-order structures to allow binding to its interacting proteins, and *Neat1* may have a conserved function in myogenesis between human and mouse.

**Fig. 8:**
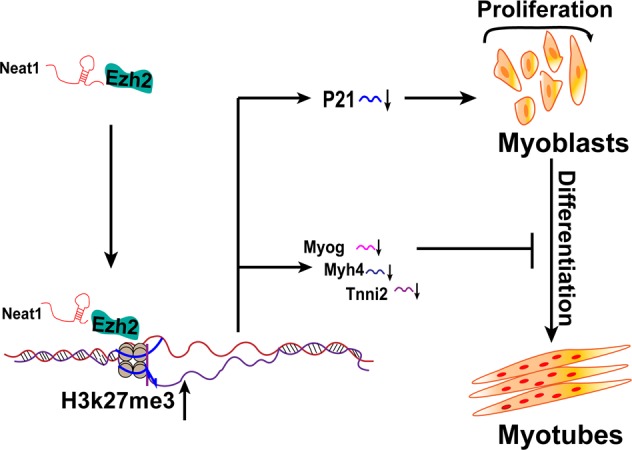
Schematic model of *Neat1* regulation in myogenesis. In proliferating myoblasts, *Neat1* guides Ezh2 to the *P21* promoter and inhibits *P21* expression, leading to the promotion of myoblast proliferation. Upon differentiation, *Neat1* recruits Ezh2 to inhibit the expression of muscle-specific genes, such as *Myog*, *Myh4*, and *Tnni2*, and suppresses myogenic differentiation

In conclusion, our findings provide a novel function of *Neat1* in muscle development and regeneration. We demonstrated that *Neat1* promoted myoblast proliferation and repressed myogenic differentiation, and knockdown of *Neat1* promoted muscle growth but impaired muscle regeneration. Mechanistically, *Neat1* recruited Ezh2 to increase the level of H3k27me3 binding at the *P21* promoter, leading to repression of *P21* expression and promotion of myoblast proliferation. Meanwhile, *Neat1* inhibited the expression of muscle-specific genes, such as *Myog*, *Myh4*, and *Tnni2*, by recruiting Ezh2 to target gene promoters and thereby suppressing myogenic differentiation (Fig. 8).

## Supplementary information

Supplementary Figures

Table S1

Table S2

Table S3

Table S4
