# Supplementary material for: Long noncoding RNA Neat1 modulates myogenesis by recruiting Ezh2
Source: Cell Death Dis. 2019 Jun 26;10(7):505. doi: 10.1038/s41419-019-1742-7 (PMC6594961; doi:10.1038/s41419-019-1742-7)
Supplement: Supplementary file 1 — Supplementary Figures [file 41419_2019_1742_MOESM1_ESM.docx]

**
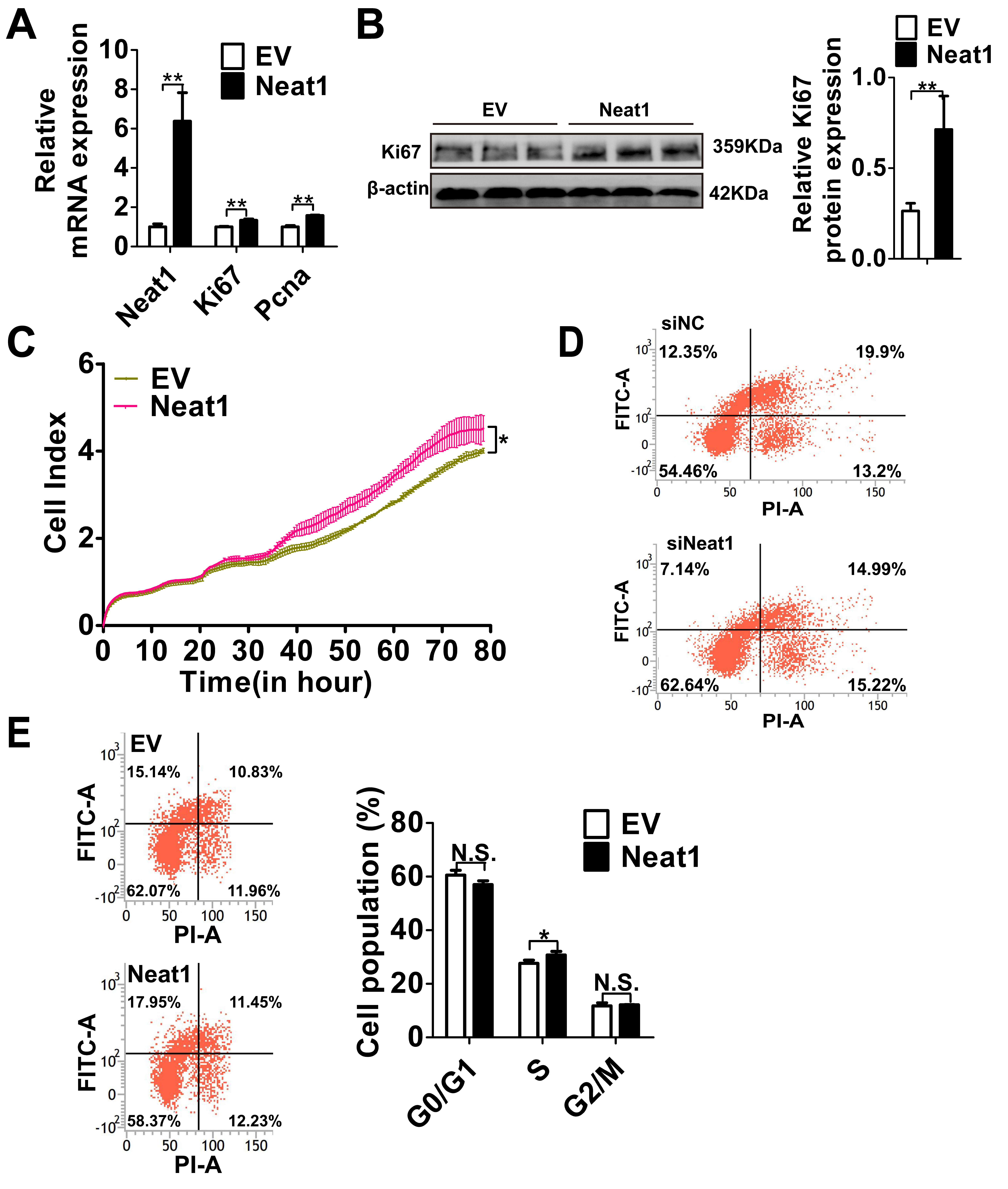
**

**Fig. S1** ***Neat1* promotes the proliferation of C2C12 myoblasts. a** qPCR results showing that the mRNA expression of *Ki67* and *Pcna* was significantly increased by *Neat1* overexpression. **b** Western blotting analysis showing that Ki67 protein was significantly increased by *Neat1* overexpression. The Ki67 protein level was quantified using ImageJ software. **c** The RTCA xCELLigence result demonstrating that cell growth dynamics was significantly increased after *Neat1* overexpression. **d** **Representative picture of EdU-propidium iodide (PI) flow cytometry showing that the proportion of cells in S phase was significantly decreased by *Neat1* knockdown.** **e** **EdU-PI flow cytometry result showing that the proportion of cells in S phase was significantly increased by *Neat1* overexpression.** Relative mRNA and protein level were normalized to those of β-actin. All values represent the mean ± s.d. of three independent experiments. * p < 0.05, ** p < 0.01, N.S. indicates not signiﬁcant.


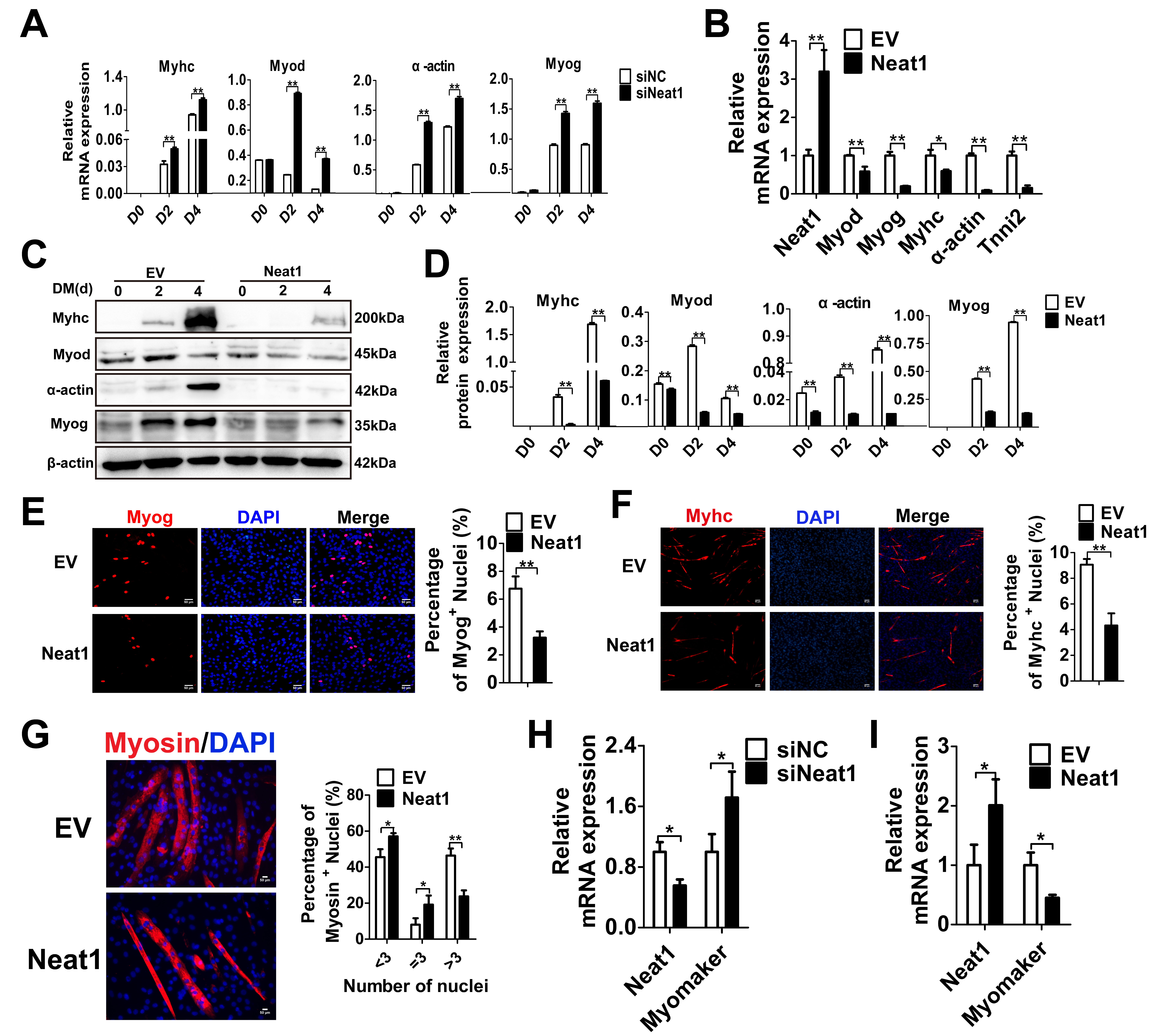


**Fig. S2** ***Neat1* inhibits myogenic differentiation and fusion.** **a** **The quantification results of Myod, Myog, Myhc, and α-actin protein expression of Fig. 2f. b** qPCR results showing that the mRNA expression of *Myod*, *Myog*, *Myhc*, *α-actin*, and *Tnni2* was significantly decreased by *Neat1* overexpression in C2C12 cells on day 2 post-differentiation. **c-d** Western blotting analysis showing that the protein expression of Myod, Myog, Myhc, and α-actin was significantly decreased by *Neat1* overexpression in C2C12 cells on days 0, 2, and 4 post-differentiation (**c**). The protein expression of Myod, Myog, Myhc, and α-actin was quantified using ImageJ software (**d**). **e** Immunofluorescence staining showing that Myog protein expression was significantly inhibited by *Neat1* overexpression on day 2 post-transfection. Cell nuclei were stained with DAPI. The number of Myog-positive cells was quantified using ImageJ software. **f** Immunofluorescence staining of Myhc showing that *Neat1* overexpression significantly inhibited C2C12 differentiation on day 3 post-transfection. The number of Myhc-positive cells was quantified using ImageJ software. **g** **Immunofluorescence staining of myosin in C2C12 cells differentiated for 5 days showing that *Neat1* overexpression significant reduced the fusion rato of myoblasts.** **h** **qPCR results showed that *Neat1* knockdown increased fusion marker gene *Myomaker* expression. i** **qPCR results showed that *Neat1* overexpression decreased *Myomaker* expression.** Relative RNA and protein levels were normalized to those of β-actin. All values represent the mean ± s.d. of three independent experiments. * p < 0.05, ** p < 0.01.

**Fig. S3** ***Neat1* physically interacts with Ezh2 to affect myoblasts priliferation and differentiation.**  **a** The *Ezh2* mRNA level was monitored by qPCR after *Neat1* knockdown or overexpression. Neither *Neat1* knockdown nor overexpression altered *Ezh2* mRNA expression. **b** The Ezh2 protein level was examined by Western blotting after *Neat1* knockdown or overexpression and quantified using ImageJ. Neither *Neat1* knockdown nor overexpression affected the Ezh2 protein level. **c** The *Neat1* mRNA level was examined by qPCR after *Ezh2* knockdown or overexpression. Neither *Ezh2* knockdown nor overexpression affected *Neat1* expression. **d** RIP assays were performed in C2C12 cells using Suz12 antibodies. **e** **Representative picture of EdU staining results showing that overexpression of F3, but not the other fragments, and full-length *Neat1* significantly promoted C2C12 cell proliferation. f, g Representative pictures of Myog and Myhc immunofluorescence staining results showing that overexpression of F3, but not the other fragments, with full-length *Neat1* significantly inhibited Myog protein expression (f) and C2C12 cell differentiation (g).** **h** **Identification of *Neat1*-binding proteins by RNA pulldown experiment. The proteins pulled down by *Neat1* and *Neat1*-AS (the antisense RNA of *Neat1*, used as a negative control) were separated by SDS-PAGE and performed with silver staining. The differentially expressed bands in the *Neat1*-binding group were used for mass spectrometry (denoted in the black box). U1 was used as negative controls.** Relative RNA and protein levels were normalized to those of β-actin. All values represent the mean ± s.d. of three independent experiments. * p < 0.05, N.S. indicates not signiﬁcant.


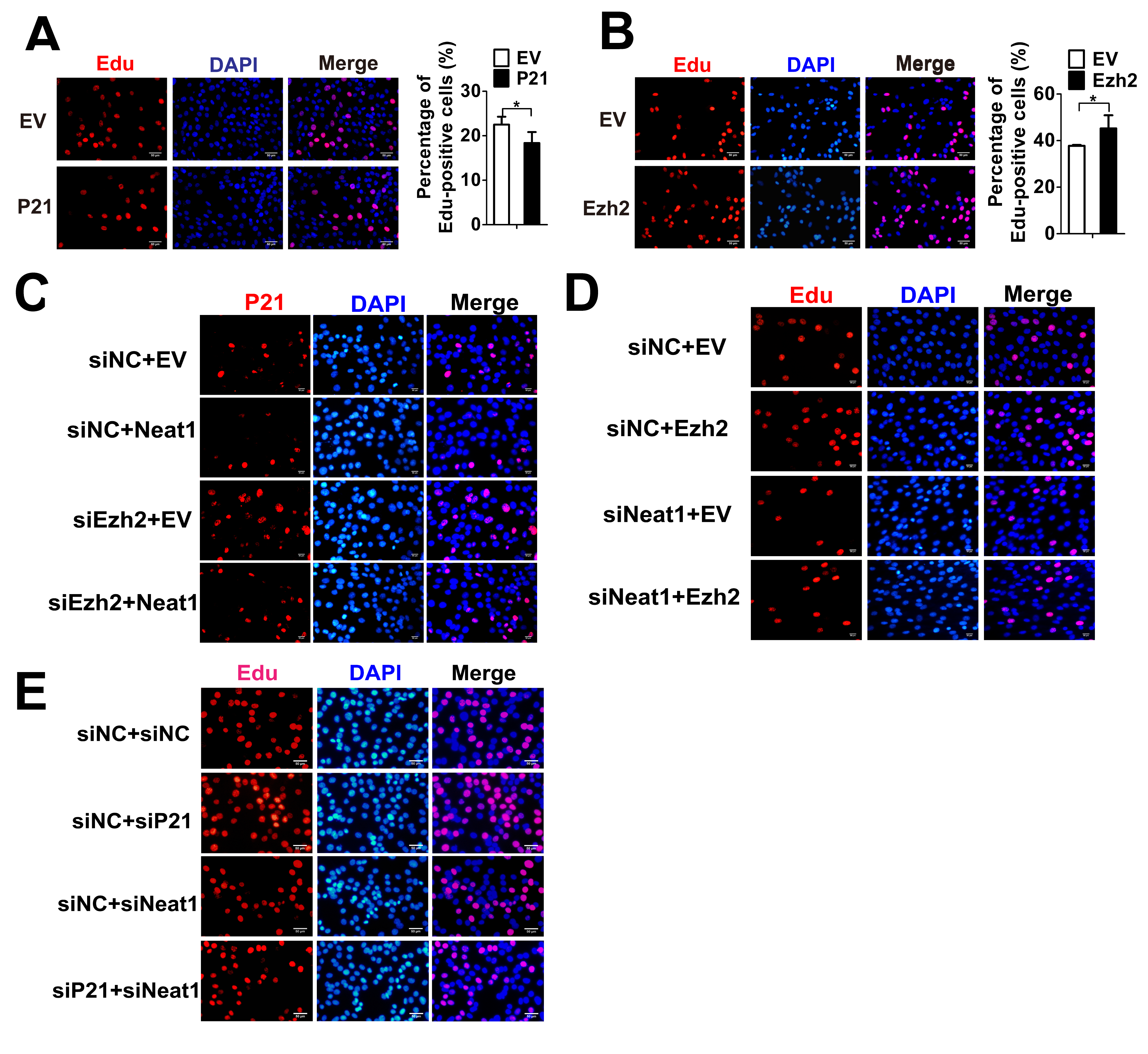


**Fig. S4 *Neat1* promotes C2C12 proliferation through Ezh2-mediated P21 inhibition. a-b** **Representative picture of EdU staining showed that the overexpression of *P21* inhibited myoblast proliferation** (**a**), **whereas *Ezh2* overexpression promoted myoblast proliferation** (**b**). **The percentage of EdU^+^ cells was quantified using ImageJ software. c Representative picture of** **P21 immunofluorescence staining results showing that overexpression of *Neat1* inhibited P21 protein expression, but had no significant effect on P21 expression after co-transfection with *Ezh2* siRNA fragment.** **d Representative picture of EdU staining results showed that *Neat1* knockdown inhibited myoblast proliferation. After co-transfected with *Ezh2* expression vector, *Neat1* knockdown can not inhibit myoblast proliferation. e Representative picture of EdU staining showed that *Neat1* Knockdown significantly reduced the percentage of EdU^+^ cells. After transfection with *P21* siRNA fragment, *Neat1* knockdown did not reduce the number of EdU^+^ cells compared with the control. All values represent the mean ± s.d. of three independent experiments. * p < 0.05.**


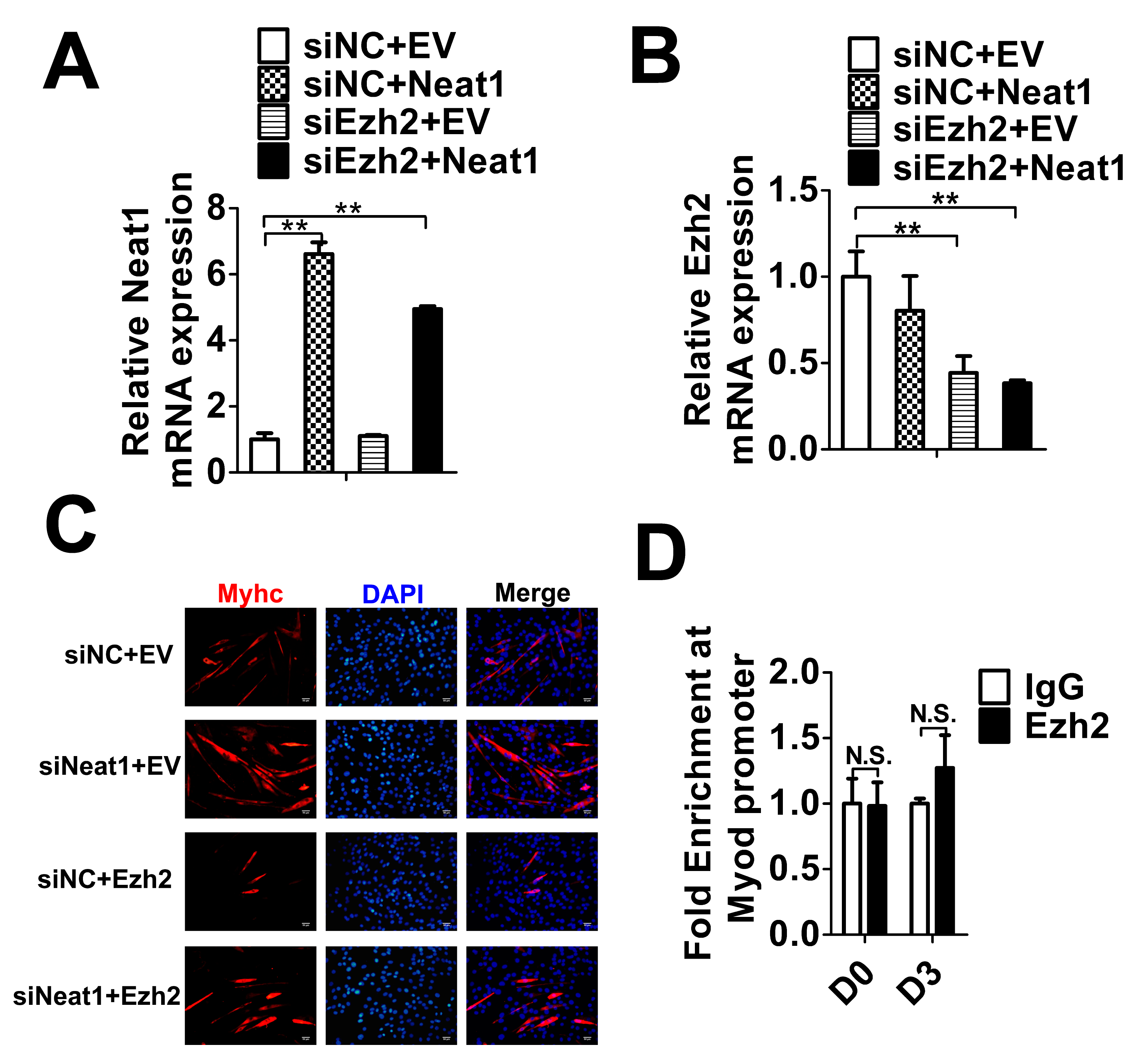


**Fig. S5 The expression of *Neat1* and *Ezh2* were examined following their co-transfection into C2C12 cells; *Neat1* affects Myhc expression by Ezh2; Ezh2 does not bind to the *Myod* promoter.** **a-b** *Neat1* expression vector and *Ezh2* siRNA fragment were co-transfected into C2C12 myoblasts. The *Neat1* (**a**) mRNA expression level was significantly increased, and the *Ezh2* (**b**) mRNA expression level significantly decreased on day 3 post-differentiation. **c** **Representative picture of Myhc immunofluorescence staining results showing that *Neat1* knockdown alone increased Myhc protein expression but had no effects when co-transfected with Ezh2 expression vector.**  **d** ChIP assays were performed after C2C12 cells differentiated at 0 and 3 days. Endogenous Ezh2 did not bind to the promoter of *Myod*. All values represent the mean ± s.d. of three independent experiments. ** p < 0.01, N.S. indicates not signiﬁcant.


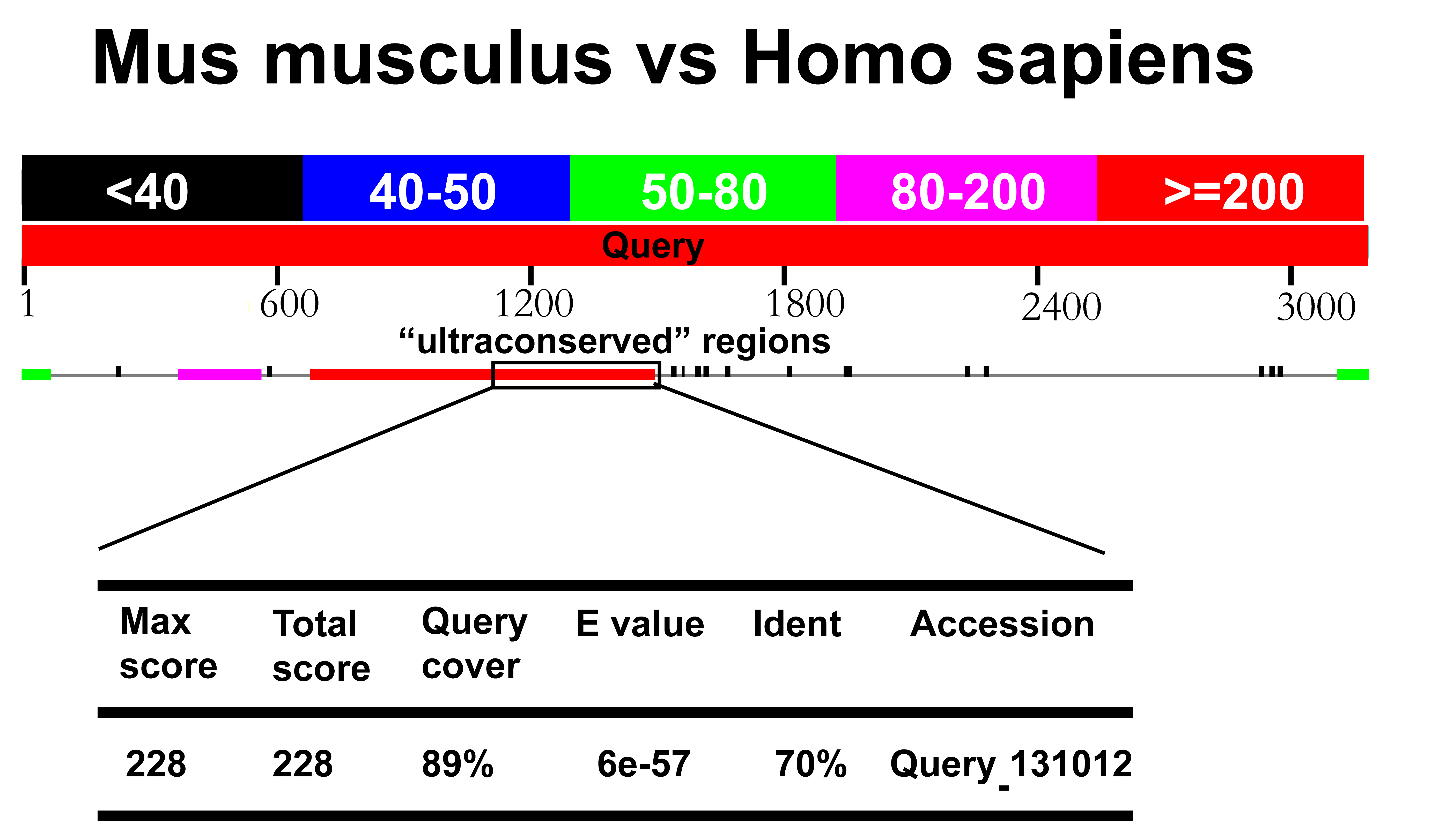


**Fig. S6** **The “ultraconserved” regions of *Neat1* between mice and humans.** The core domain sequence of *Neat1* responsible for binding to Ezh2 (the 1001–1540 bp fragment denoted in the black box above) was aligned to that of human *NEAT1* using BLAST. This region is conserved between mouse and human.
