# Supplementary material for: Long noncoding RNA Neat1 modulates myogenesis by recruiting Ezh2
Source: Cell Death Dis. 2019 Jun 26;10(7):505. doi: 10.1038/s41419-019-1742-7 (PMC6594961; doi:10.1038/s41419-019-1742-7)
Supplement: Supplementary file 2 — Table S1 [file 41419_2019_1742_MOESM2_ESM.docx]

**Table S1. Primers used for plasmid construction**

| **Primer name** | **Primer sequence(5’-3’)** |
| --- | --- |
| *Neat1* full-length sequence | F: AGGAGTTAGTGACAAGGAG |
|  | R: GAAGCTTCAATCTCAAACCTTTA |
| *Ezh2*-cds | F: TCGCGTCCGACACCCAG |
|  | R: TGCCCACAGTACTCAAGGTTCC |
| *P21* cds | F: GCCAGCAGAATAAAAGGTGCC |
|  | R: AGTAGGACTGTTCCTCCGGT |
| *Neat1* 1-500bp fragment | F: AGGAGTTAGTGACAAGGAG |
|  | R: GGCCTCATCCCCAGGTGGGT |
| *Neat1* 501-1000bp fragment | F: TGGTCTTATGGAAGGTGGA |
|  | R: ATGGCTGTGAGCCCACCCCA |
| *Neat1* 1001-1540bp fragment | F: GTTTCCGGTCACCCAAATG |
|  | R: GGTCACTGGGGTCCCGATC |
| *Neat1* 1541-3190bp fragment | F: TCGCCCACCTTCCCTGCT |
|  | R: GAAGCTTCAATCTCAAACCTTTA |
