# Supplementary material for: Long noncoding RNA Neat1 modulates myogenesis by recruiting Ezh2
Source: Cell Death Dis. 2019 Jun 26;10(7):505. doi: 10.1038/s41419-019-1742-7 (PMC6594961; doi:10.1038/s41419-019-1742-7)
Supplement: Supplementary file 3 — Table S2 [file 41419_2019_1742_MOESM3_ESM.docx]

**Table S2. Primers used for qPCR**

| **Gene or Primer name** | **Primer sequence(5’-3’)** |
| --- | --- |
| *Neat1* | F: GGGAAGGGTGACATTGAAAA |
|  | R: CTCCCCAGCTTCACTTCTTG |
| *Myog* | F: CCATCCAGTACATTGAGCGCCTACA |
|  | R: ACGATGGACGTAAGGGAGTGCAGAT |
| *Myhc* | F: CAAGTCATCGGTGTTTGTGG |
|  | R: TGTCGTACTTGGGCGGGTTC |
| *Myod* | F: CGAGCACTACAGTGGCGACTCAGAT |
|  | R: GCTCCACTATGCTGGACAGGCAGT |
| *Pax7* | F: GCTACCAGTACAGCCAGTATG |
|  | R: GTCACTAAGCATGGGTAGATG |
| *Ki67* | F: ATCATTGACCGCTCCTTTAGGT |
|  | R: GCTCGCCTTGATGGTTCCT |
| *Pcna* | F: TTTGAGGCACGCCTGATCC |
|  | R: GGAGACGTGAGACGAGTCCAT |
| *α-actin* | F: CACCAGGGTGTCATGGTAGG |
|  | R: TGGTACGGCCGGAAGCATAG |
| *Tnni2* | F: CCTGAAGAGTGTGATGCTCCA |
|  | R: CCCGTTCCTTCTCAGTGTCTT |
| *eMyhc* | F: TATCAGAGTGAGGAGGACAG |
|  | R: TCGCTTTCATGGACCACCAT |
| *β-actin* | F: GCCTCACTGTCCACCTTCCA |
|  | R: AGCCATGCCAATGTTGTCTCTT |
| *Gapdh* | F: AGAACATCATCCCTGCATCC |
|  | R: GGTCCTCAGTGTAGCCCAAG |
| *Ezh2* | F: CCGCTTTCCTGGATGTCG |
|  | R: GTTGCCCTTTCGGGTTGC |
| ***Myomaker*** | **F: ATCGCTACCAAGAGGCGTT** |
|  | **R: CACAGCACAGACAAACCAGG** |
| ***U1*** | **F: GGGAGATACCATGATCACGAAGGT** |
|  | **R: CCACAAATTATGCAGTCGAGTTTCCC** |
