# Supplementary material for: Long noncoding RNA Neat1 modulates myogenesis by recruiting Ezh2
Source: Cell Death Dis. 2019 Jun 26;10(7):505. doi: 10.1038/s41419-019-1742-7 (PMC6594961; doi:10.1038/s41419-019-1742-7)
Supplement: Supplementary file 4 — Table S3 [file 41419_2019_1742_MOESM4_ESM.docx]

**Table S3. Primers used for ChIP or ChIRP**

| **Gene or Primer name** | **Primer sequence(5’-3’)** |
| --- | --- |
| *Myog* | F: GAGTGGTCCTGATGTGGTAGTGG |
|  | R: GCCGTCGGCTGTAATTTGAT |
| *Myh4* | F: CACCCAAGCCGGGAGAAACAGCC |
|  | R: GAGGAAGGACAGGACAGAGGCACC |
| *Tnni2* | F: GTGAGGCCCAGCCCATCTTC |
|  | R: CATTTTCCTGCTTGTGTGTC |
| *P21* | F: TAACGCGCGCCGGTTCTA |
|  | R: CTGCGCCTGACTCCAATTCC |
| *Myod* | F: CTGATACTGGCGGTGCTGAT |
|  | R: GAAGGTTGCTGGTTATGCTA |
| *Gapdh* | F: GAATGCCTTTTCTCCCTTCC |
|  | R: GAGCCAGGGACTCTCCTTTT |
