# Supplementary material for: Long noncoding RNA Neat1 modulates myogenesis by recruiting Ezh2
Source: Cell Death Dis. 2019 Jun 26;10(7):505. doi: 10.1038/s41419-019-1742-7 (PMC6594961; doi:10.1038/s41419-019-1742-7)
Supplement: Supplementary file 5 — Table S4 [file 41419_2019_1742_MOESM5_ESM.docx]

**Table 4: Identification of Neat1-binding proteins by mass spectrometry**

| Protein | Description | Gene | MW [kDa] |
| --- | --- | --- | --- |
| Q71LX8 | Heat shock protein 84b | Hsp90ab1 | 83.2 |
| Q8CAQ8 | MICOS complex subunit Mic60 | Immt | 83.8 |
| Q11011 | Puromycin-sensitive aminopeptidase | Npepps | 103.3 |
| Q3TI61 | 26S proteasome non-ATPase regulatory subunit 2 | Psmd2 | 100.2 |
| P58252 | Elongation factor 2 | Eef2 | 95.3 |
| Q3TVV6 | Uncharacterized protein | Hnrnpu | 87.9 |
| P07901 | Heat shock protein HSP 90-alpha | Hsp90aa1 | 84.7 |
| Q3TRW3 | Staphylococcal nuclease domain-containing protein | Snd1 | 102 |
| Q3UAD6 | Heat shock protein 90kDa beta (Grp94), member 1 | Hsp90b1 | 92.4 |
| Q8C605 | ATP-dependent 6-phosphofructokinase | Pfkp | 85.5 |
| P48678 | Prelamin-A/C | Lmna | 74.2 |
| Q3V3R1 | Monofunctional C1-tetrahydrofolate synthase, mitochondrial | Mthfd1l | 105.7 |
| Q3UZH3 | Uncharacterized protein (Fragment) | Mthfd1 | 101.4 |
| E9QB02 | Methionine--tRNA ligase, cytoplasmic | Mars | 102.3 |
| Q3ULC7 | Uncharacterized protein | Ddx21 | 93.5 |
| Q80Y09 | Pdcd6ip protein | Pdcd6ip | 96.3 |
| Q91YR7 | Pre-mRNA-processing factor 6 | Prpf6 | 106.7 |
| Q3V122 | DNA replication licensing factor MCM7 | Mcm7 | 81.3 |
| O35286 | Pre-mRNA-splicing factor ATP-dependent RNA helicase DHX15 | Dhx15 | 90.9 |
| Q3TJ43 | Vacuolar protein sorting-associated protein 35 | Vps35 | 91.6 |
| Q8R1F1 | Niban-like protein 1 | Fam129b | 84.8 |
| Q01853 | Transitional endoplasmic reticulum ATPase | Vcp | 89.3 |
| Q8BJS4 | SUN domain-containing protein 2 | Sun2 | 81.6 |
| Q3TWN8 | Delta-1-pyrroline-5-carboxylate synthase | Aldh18a1 | 87.2 |
| Q8BJ71 | Nuclear pore complex protein Nup93 | Nup93 | 93.2 |
| Q99KI0 | Aconitate hydratase, mitochondrial | Aco2 | 85.4 |
| Q3TZ32 | Uncharacterized protein | Aars | 106.8 |
| O55029 | Coatomer subunit beta | Copb2 | 102.4 |
| Q9Z1Z0 | General vesicular transport factor p115 | Uso1 | 106.9 |
| Q7TPR4 | Alpha-actinin-1 | Actn1 | 103 |
| G3X8Y3 | N-alpha-acetyltransferase 15, NatA auxiliary subunit | Naa15 | 101 |
| A0A1L1STE4 | Interleukin enhancer-binding factor 3 | Ilf3 | 97.4 |
| Q569X8 | Nuclear pore complex protein | Nup107 | 106.6 |
| Q3TXF9 | Sodium/potassium-transporting ATPase subunit alpha | Atp1a1 | 112.9 |
| Q8VIJ6 | Splicing factor, proline- and glutamine-rich | Sfpq | 75.4 |
| Q3TXS7 | 26S proteasome non-ATPase regulatory subunit 1 | Psmd1 | 105.7 |
| F8WIV5 | Dynamin-2 | Dnm2 | 98 |
| Q3TMP1 | General transcription factor IIIC, polypeptide 3 | Gtf3c3 | 100.6 |
| Q8CGK3 | Lon protease homolog, mitochondrial | Lonp1 | 105.8 |
| Q8CIJ3 | Eukaryotic translation initiation factor 3 subunit B | Eif3b | 108.9 |
| P17710 | Hexokinase-1 | Hk1 | 108.2 |
| Q3U8W9 | Uncharacterized protein | Hnrnpr | 70.8 |
| Q8K009 | Mitochondrial 10-formyltetrahydrofolate dehydrogenase | Aldh1l2 | 101.5 |
| E9Q3X0 | Major vault protein | Mvp | 96.8 |
| Q9QZE5 | Coatomer subunit gamma-1 | Copg1 | 97.5 |
| Q8K363 | ATP-dependent RNA helicase DDX18 | Ddx18 | 74.1 |
| Q5EBP9 | Tripartite motif-containing 28 | Trim28 | 88.8 |
| P97311 | DNA replication licensing factor MCM6 | Mcm6 | 92.8 |
| Q9JIF7 | Coatomer subunit beta | Copb1 | 107 |
| Q61768 | Kinesin-1 heavy chain | Kif5b | 109.5 |
| Q69ZD1 | MKIAA1699 protein (Fragment) | Exoc4 | 110.7 |
| Q5SYD0 | Unconventional myosin-Id | Myo1d | 116 |
| Q8C7S2 | Uncharacterized protein | Lima1 | 84 |
| Q6ZQ61 | MCG121979, isoform CRA_c (Fragment) | Matr3 | 95.2 |
| A2A432 | Cullin-4B | Cul4b | 110.6 |
| Q6A0E3 | MKIAA0031 protein (Fragment) | Eftud2 | 109.7 |
| E9Q8Z5 | Catenin delta-1 | Ctnnd1 | 104.9 |
| Q3UJN1 | DNA helicase | Mcm2 | 103.1 |
| A1L2Z3 | C230096C10Rik protein | Emc1 | 111.6 |
| Q6P542 | ATP-binding cassette sub-family F member 1 | Abcf1 | 94.9 |
| Q8BMS1 | Trifunctional enzyme subunit alpha, mitochondrial | Hadha | 82.6 |
| Q8CC13 | AP complex subunit beta | Ap1b1 | 104.9 |
| Q3V1Z7 | Uncharacterized protein | Ddx1 | 82.4 |
| A0A1S6GWJ8 | Uncharacterized protein | Hnrnpm | 86.4 |
| A0JNY7 | Eukaryotic translation initiation factor 4, gamma 2 | Eif4g2 | 102.1 |
| P27612 | Phospholipase A-2-activating protein | Plaa | 87.2 |
| Q01405 | Protein transport protein Sec23A | Sec23a | 86.1 |
| Q02248 | Catenin beta-1 | Ctnnb1 | 85.4 |
| Q8R1B4 | Eukaryotic translation initiation factor 3 subunit C | Eif3c | 105.5 |
| Q00PI9 | Heterogeneous nuclear ribonucleoprotein U-like protein 2 | Hnrnpul2 | 84.9 |
| Q8VHK9 | ATP-dependent DNA/RNA helicase DHX36 | Dhx36 | 113.8 |
| O08528 | Hexokinase-2 | Hk2 | 102.5 |
| A0A1B0GSU0 | Aldehyde dehydrogenase family 16 member A1 | Aldh16a1 | 84.7 |
| P70168 | Importin subunit beta-1 | Kpnb1 | 97.1 |
| G5E8C4 | MCG142017, isoform CRA_a | Tmtc3 | 104.1 |
| Q3TIN2 | Uncharacterized protein | Qars | 87.7 |
| A0A0R4J0E4 | Integrator complex subunit 7 | Ints7 | 106.8 |
| Q8BJW5 | Nucleolar protein 11 | Nol11 | 80.8 |
| P48722 | Heat shock 70 kDa protein 4L | Hspa4l | 94.3 |
| Q3UKP5 | Uncharacterized protein | Xab2 | 99.8 |
| Q4FZC9 | Nesprin-3 | Syne3 | 112 |
| Q8VDC3 | Cytoplasmic aconitase | aco1 | 99 |
| Q99MR8 | Methylcrotonoyl-CoA carboxylase subunit alpha, mitochondrial | Mccc1 | 79.3 |
| G3X9F1 | GPI ethanolamine phosphate transferase 1 | Pign | 105 |
| A2BE28 | Ribosomal biogenesis protein LAS1L | Las1l | 89.4 |
| Q6KAR6 | Exocyst complex component 3 | Exoc3 | 86.4 |
| A0A0R4IZY0 | Thimet oligopeptidase | Thop1 | 78 |
| Q61033 | Lamina-associated polypeptide 2, isoforms alpha/zeta | Tmpo | 75.1 |
| Q3TCH7 | Cullin-4A | Cul4a | 87.7 |
| Q8K2J0 | 1-phosphatidylinositol 4,5-bisphosphate phosphodiesterase delta-3 | Plcd3 | 88.6 |
| Q91W86 | Vacuolar protein sorting-associated protein 11 homolog | Vps11 | 107.7 |
| Q569Z6 | Thyroid hormone receptor-associated protein 3 | Thrap3 | 108.1 |
| P33146 | Cadherin-15 | Cdh15 | 85.6 |
| D3Z0M9 | DEAD (Asp-Glu-Ala-Asp) box polypeptide 23 | Ddx23 | 95.4 |
| Q99KC8 | von Willebrand factor A domain-containing protein 5A | Vwa5a | 87.1 |
| A0A1L1SV25 | Alpha-actinin-4 | Actn4 | 107 |
| Q9ESL4 | Mitogen-activated protein kinase kinase kinase 20 | Map3k20 | 91.7 |
| Q6P1Y9 | Exocyst complex component 1 | Exoc1 | 102.6 |
| Q14CH7 | Alanine--tRNA ligase, mitochondrial | Aars2 | 106.7 |
| A0A068EW80 | Promyelocytic leukemia | Pml | 98.2 |
| Q571A2 | MKIAA4106 protein (Fragment) | Cul2 | 87.1 |
| Q5BKS5 | Hook homolog 3 (Drosophila) | Hook3 | 83.4 |
| Q8BML1 | [F-actin]-monooxygenase MICAL2 | Mical2 | 110 |
| Q8VE19 | GATOR complex protein MIOS | Mios | 98.3 |
| Q3TUQ5 | Pinin | Pnn | 82.5 |
| P51660 | Peroxisomal multifunctional enzyme type 2 | Hsd17b4 | 79.4 |
| D6Q0F5 | Cytoplasmic dynein intermediate chain 2 isoform 2.1 | Dync1i2 | 73.3 |
| Q8CIM8 | Integrator complex subunit 4 | Ints4 | 108.1 |
| Q8CHW4 | Translation initiation factor eIF-2B subunit epsilon | Eif2b5 | 80 |
| A2A7Q5 | Prolyl 3-hydroxylase 1 | P3h1 | 84.4 |
| Q8CJG0 | Protein argonaute-2 | Ago2 | 97.2 |
| Q61584 | Fragile X mental retardation syndrome-related protein 1 | Fxr1 | 76.2 |
| P56695 | Wolframin | Wfs1 | 100.5 |
| Q8BFY9 | Transportin-1 | Tnpo1 | 102.3 |
| Q3TDD9 | Protein phosphatase 1 regulatory subunit 21 | Ppp1r21 | 88.3 |
| Q80W68 | Kin of IRRE-like protein 1 | Kirrel1 | 87.1 |
| Q80TA6 | Myotubularin-related protein 12 | Mtmr12 | 85.5 |
| Q9EP71 | Ankycorbin | Rai14 | 108.8 |
| B6ZHD0 | Erythrocyte protein band 4.1-like 2 | Epb41l2 | 109.8 |
| Q6P5B5 | Fragile X mental retardation syndrome-related protein 2 | Fxr2 | 74.2 |
| Q9WTR1 | Transient receptor potential cation channel subfamily V member 2 | Trpv2 | 85.9 |
| D3YZV8 | Coiled-coil domain-containing protein 8 homolog | Ccdc8 | 73.9 |
